# Spatiotemporal dynamics of culturable endophytic microorganisms in *Peucedanum praeruptorum* Dunn and screening for plant growth-promoting strains

**DOI:** 10.3389/fpls.2026.1811872

**Published:** 2026-04-27

**Authors:** Bin Deng, Yue Dong, Yiping Qin, Qi Huang, Han Luo, Congbin Liu, Dongmei Xie

**Affiliations:** 1 School of Pharmacy, Anhui University of Chinese Medicine, Hefei, China; 2 Institute of Traditional Chinese Medicine (TCM) Resources Protection and Development, Anhui Academy of Chinese Medicine, Hefei, China

**Keywords:** community structure, endophytes, *Peucedanum praeruptorum* Dunn, plant growth-promoting traits, spatiotemporal dynamics

## Abstract

Peucedani Radix is the dried root of *Peucedanum praeruptorum* Dunn (Apiaceae), which is traditionally used for relieving cough and asthma, eliminating phlegm, dispersing wind, and clearing heat. Previous studies on endophytes associated with *Peucedanum praeruptorum* were largely restricted to specific plant compartments and lacked a comprehensive assessment of endophytic communities across different organs and developmental stages of *P. praeruptorum*. To investigate the spatiotemporal distribution of endophytic microorganisms in *Peucedanum praeruptorum* Dunn and assess their potential as plant growth-promoting (PGP) resources, root, stem, and leaf samples were collected from September to December 2024. Endophytic microbial community structures were analyzed using high-throughput sequencing, combined with culture-dependent isolation to screen functional strains, and the growth-promoting strains were co-cultivated with *Arabidopsis thaliana* and *P. praeruptorum* to verify their growth-promoting effects. A total of 2,592 fungal and 6,705 bacterial amplicon sequence variants (ASVs) were obtained, with 173 fungal and 39 bacterial strains successfully isolated. The endophytic microbial communities exhibited significant spatiotemporal heterogeneity, with the highest diversity observed in September. Additionally, community composition differed markedly among tissues, particularly between roots and aboveground organs (stems and leaves). Proteobacteria and Ascomycota were identified as the dominant bacterial and fungal phyla, respectively. Several strains possessing PGP traits, including phosphate solubilization, and potassium solubilization, were successfully screened. The validation results of growth-promoting effects indicated that some strains could significantly promote the growth of both *A. thaliana* and *P. praeruptorum*. This study elucidates the community characteristics and functional potential of endophytes in *P. praeruptorum*, providing a theoretical basis and valuable microbial resources for ecological cultivation and microbial inoculant development.

## 1 Introduction

Endophytes are a distinct group of microorganisms that colonize the internal tissues of healthy plants and establish mutualistic symbiotic relationships with their hosts throughout all or most of their life cycle without causing apparent disease symptoms (Upadhyay and Khandelwal, 2025). Studies of plant-associated microbial diversity have shown that endophytes mainly include fungi, bacteria, and actinomycetes (Huang and Zhao, 2023). These microorganisms form multifaceted symbioses with host plants and can effectively promote plant growth and enhance resistance to diseases and abiotic stresses through multiple mechanisms, such as biological nitrogen fixation, potassium solubilization, phosphate solubilization, siderophore production, phytohormone synthesis, and the production of 1-aminocyclopropane-1-carboxylate (ACC) deaminase (Dudeja et al., 2012; Paula et al., 2023; Cen et al., 2024; Chanda et al., 2024; Jan et al., 2025; Lopez et al., 2025; Pandey and Saharan, 2025). In medicinal plants, endophytes have attracted considerable attention due to their ability to directly produce bioactive compounds identical or similar to those of their host plants, or indirectly modulate the host’s biosynthetic pathways, thereby influencing medicinal material quality and pharmacological efficacy (Harshitha et al., 2023; Pacheco-Ramírez et al., 2025; Nishant and Chandra, 2026).

Peucedani radix is the dried root of *Peucedanum praeruptorum* Dunn (Apiaceae), which is traditionally used for relieving cough and asthma, eliminating phlegm, dispersing wind, and clearing heat (Commission, 2020). Modern pharmacological studies have demonstrated that *P. praeruptorum* is rich in diverse chemical constituents, including coumarins and glycosides (Chen et al., 2021), and exhibits a wide range of biological activities, such as antiarrhythmic, anti-heart failure, antihypertensive, anti-inflammatory, analgesic, and antipyretic effects (Yu et al., 2012; Sun et al., 2013; Lin et al., 2020; Wang et al., 2024), highlighting its high medicinal value. With the continuous increase in market demand, wild resources of Peucedani radix have been overharvested, making artificial cultivation the primary source of medicinal materials. However, factors such as growth environment, cultivation practices, and harvesting time may significantly affect the quality of *P. praeruptorum* roots (Xu et al., 2023; Shi et al., 2024; Ge et al., 2025).

The structure of endophytic microbial communities is not static but is shaped by complex interactions among plant tissues, geographic location, and seasonal variation (Tang et al., 2024; Li et al., 2025; Yan et al., 2025). Previous studies on endophytes associated with *P. praeruptorum* have mainly focused on rhizosphere soil microorganisms, the composition and diversity of root-associated endophytes, and the effects of exogenous plant growth-promoting (PGP) rhizobacteria on plant growth and physiological traits (Zhang et al., 2023). These studies were largely restricted to specific plant compartments and lacked a comprehensive assessment of endophytic communities across different organs and developmental stages of *P. praeruptorum.* In the present study, high-throughput sequencing (HTS) was employed to systematically characterize the endophytic microbial community structures in roots, stems, and leaves of *P. praeruptorum* at different growth stages. In parallel, strains exhibiting phosphate-solubilizing, and potassium-solubilizing capabilities were isolated and purified, and the intensities of these functional traits were quantitatively evaluated. Meanwhile, by co-cultivating strains with growth-promoting traits with *Arabidopsis thaliana* (aseptic *in vitro* seedlings) and *P. praeruptorum*, the *in vivo* growth-promoting characteristics of the strains were clarified through the determination of physiological and biochemical indicators. This work contributes to a deeper understanding of the spatiotemporal distribution patterns of endophytic microbial communities in *P. praeruptorum* and their interactions with host growth and development, and provides both a theoretical foundation and high-quality microbial resources for the future application of microbial inoculants to regulate plant growth and to establish sustainable, eco-friendly cultivation systems.

## 2 Materials and methods

### 2.1 Plant materials

Root, stem, and leaf samples of *P. praeruptorum* Dunn were collected in September, October, November, and December 2024 from Nanji Township, Ningguo City, Anhui Province, China(119.0195° E, 30.4056° N) (**Table 1**). At each sampling site, ten healthy plants showing no visible disease symptoms and uniform growth were randomly selected, and three biological replicates were established. All samples were placed into sterile sampling bags, labeled, sealed, and stored in foam containers with dry ice, and immediately transported to the laboratory for further processing. All plant samples were thoroughly rinsed with running tap water to remove surface soil particles and subsequently surface-sterilized in a laminar flow hood following a standard protocol: washing with sterile water for 30 s, immersion in 30 mL of 75% (v/v) ethanol for 1 min, treatment with 20 mL of 2% (w/v) NaClO (reagent-grade) for 3 min, immersion in 30 mL of 75% (v/v) ethanol for 1 min, followed by four rinses with sterile water. The sterilized tissues were blotted dry with sterile filter paper and weighed, and transferred into sterile cryovials (approximately 2 g per vial), labeled, and stored at -80 °C until further analysis. To verify the effectiveness of surface sterilization, 100 μL of the final rinse water were spread onto potato dextrose agar (PDA) plates and incubated at 28 °C for 7 days. The absence of microbial growth on the plates confirmed successful surface sterilization of the plant tissues.

**Table 1:** Sampling sites and sample information.

Sample ID			Sampling date
Root	Stem	Leaf	
RA	SA	LA	2024.09.24
RB	SB	LB	2024.10.24
RC	SC	LC	2024.11.24
RD	SD	LD	2024.12.23

R, S, and L represent root, stem, and leaf tissues, respectively; A, B, C, and D correspond to the samples collected in September, October, November, and December.

### 2.2 DNA extraction, PCR amplification, and HTS

Three independent biological replicates per sample group established in Section 2.1 at each time were used for DNA extraction and subsequent high-throughput sequencing analysis. Total genomic DNA of the microbial communities was extracted using the E.Z.N.A.^®^ Soil DNA Kit (Omega Bio-tek, Norcross, GA, USA) according to the manufacturer’s instructions. The quality of the extracted DNA was assessed by electrophoresis on a 1% (w/v) agarose gel, and DNA concentration and purity were determined using a NanoDrop 2000 spectrophotometer (Thermo Scientific, USA). For fungal community analysis, the internal transcribed spacer (ITS) region was amplified using the primer pair ITS1F (5′-CTTGGTCATTTAGAGGAAGTAA-3′) and ITS2R (5′-GCTGCGTTCTTCATCGATGC-3′) (Sadiku and Shuaib, 2025). For bacterial community analysis, the V5–V7 hypervariable regions of the 16S rRNA gene were amplified using the primer pair 799F (5′-AACMGGATTAGATACCCKG-3′) and 1193R (5′-ACGTCATCCCCACCTTCC-3′) (Basit et al., 2025). PCR was performed in 20 μL reactions containing 12.5 μL of 2× Taq Mix, 0.8 μL of each forward and reverse primer (5 μmol·L^-^¹), 1 μL of template DNA (10 ng·μL^-^¹), and nuclease-free water to volume. The PCR cycling conditions were as follows: initial denaturation at 95 °C for 3 min; 40 cycles of denaturation at 95 °C for 30 s, annealing at 55 °C for 30 s, and extension at 72 °C for 45 s; followed by a final extension at 72 °C for 10 min and holding at 4 °C. PCR products were verified and excised from 2% agarose gels and purified using a DNA Gel Extraction and Purification Kit (PCR Clean-Up Kit, Yuhua, China). Purified amplicons were quantified using a Qubit 4.0 fluorometer (Thermo Fisher Scientific, USA). Sequencing libraries were constructed using the NEXTFLEX Rapid DNA-Seq Kit, and paired-end sequencing was conducted on the Illumina NextSeq 2000 platform (Shanghai Majorbio Bio-Pharm Technology Co., Ltd., China).

### 2.3 Quality control and statistical analysis of HTS data

Paired-end raw sequencing reads were quality-filtered using fastp (https://github.com/OpenGene/fastp, version 0.19.6) (Chen et al., 2018), and subsequently merged using FLASH (http://ccb.jhu.edu/software/FLASH/, version 1.2.11) (Tanja and L, 2011). The quality-controlled and merged sequences were denoised using the DADA2 plugin implemented in the QIIME2 pipeline with default parameters (Albert et al., 2012). The resulting high-resolution sequences were defined as amplicon sequence variants (ASVs) (Edgar, 2013). Sequence numbers at each processing step were summarized to evaluate data quality, and only high-quality sequences were retained for downstream analyses.

All bioinformatic and statistical analyses were performed on the Majorbio Cloud Platform (https://cloud.majorbio.com). Alpha diversity indices were calculated using mothur (Schloss et al., 2009), and differences among groups were assessed using the Kruskal-Wallis rank-sum test. Rarefaction curves were generated using R software (version 3.3.1). Beta diversity was evaluated using the weighted UniFrac distance, and intergroup differences were tested by ANOSIM. Principal coordinates analysis (PCoA) plots of endophytic fungal and bacterial communities at the genus level were visualized using R. Based on taxonomic annotation results, the top 20 most abundant taxa were selected at each taxonomic level, while the remaining taxa were grouped as “Others,” and community composition bar plots were generated using R. Functional guilds of endophytic fungi from different plant tissues were predicted using the FUNGuild database, whereas functional profiles of bacterial communities from different tissues were inferred using PICRUSt2 and the FAPROTAX database.

### 2.4 Isolation and purification of endophytic microorganisms

Endophytic fungi were isolated and purified using the tissue culture method as previously described (Li et al., 2024). Freshly collected *P. praeruptorum* plants were rinsed under running tap water for 30 min to remove surface debris. Roots and stems were cut into segments approximately 2 cm in length, and leaves were cut into squares of approximately 1 cm² using sterile scissors. Surface sterilization of different plant tissues was conducted in a laminar flow hood as follows: washing with sterile water for 30 s, immersion in 30 mL of 75% (v/v) ethanol for 1 min, treatment with 20 mL of 2% (w/v) NaClO (laboratory-grade) for 3 min, immersion in 30 mL of 75% (v/v) ethanol for 1 min, followed by four rinses with sterile water. Aliquots of the final rinse water were evenly spread onto PDA plates to serve as controls for assessing the effectiveness of surface sterilization. Surface-sterilized root, stem, and leaf segments were placed on sterile filter paper to remove excess moisture and then transferred onto PDA plates using sterile forceps. Five tissue segments were placed on each PDA plate, and five plates were prepared for each tissue type at each sampling time. The plates were incubated at 28 °C and monitored daily. When fungal hyphae emerged from the tissues, they were promptly transferred to fresh PDA plates, with a single isolate inoculated per plate, and further incubated at 28 °C to obtain pure cultures. Purified fungal isolates were preserved in 30% (v/v) glycerol at -80 °C for long-term storage.

Endophytic bacteria were isolated and purified using the plate streaking method (Feng et al., 2024). Surface sterilization was performed as described above for fungal isolation. The sterilized root, stem, and leaf tissues were homogenized in sterile mortars and pestles with an appropriate volume of sterile water to obtain tissue suspensions. The homogenates were serially diluted to 10^0^, 10^-^¹, and 10^-^², and aliquots were spread onto Luria–Bertani (LB) agar plates in triplicate. Plates were incubated at 28 °C in the dark for 7 days. The final rinse water was spread onto LB agar plates as a negative control to verify surface sterilization. After colony formation, bacterial colonies were selected based on morphological characteristics (shape, size, color, etc). and purified by repeated streaking on LB agar plates. All bacterial isolates were preserved in 50% (v/v) glycerol at -80 °C for further use.

The fungal isolation rate (IR, %) was calculated as: IR = (Ni/Nt) × 100, where Ni represents the total number of fungal isolates obtained, and Nt represents the total number of tissue segments plated.

The fungal isolation frequency (IF, %) was calculated as: IF = (n/N) × 100, where n represents the number of isolates belonging to a given taxon, and N represents the total number of endophytic fungal isolates. (IR and IF were used exclusively for fungal analysis and not applied to bacteria).

### 2.5 Molecular identification

Identification of endophytic fungi: Endophytic fungal isolates were inoculated on PDA plates at 28 °C for 7 days. An appropriate amount of mycelia and spores was scraped aseptically using a sterile inoculation loop and transferred into a sterile 2 mL microcentrifuge tube as the template for DNA extraction. Fungal genomic DNA was extracted using a modified CTAB method (Mamani et al., 2020). The extracted DNA was then used as a template for PCR amplification of the ribosomal ITS region using the universal primer pair ITS1 (5′-TCCGTAGGTGAACCTGCGG-3′) and ITS4 (5′-TCCTCCGCTTATTGATATGC-3′) (Schoch et al., 2012). PCR amplicons were examined by 1.0% (w/v) agarose gel electrophoresis and subsequently sequenced by General Biol (Anhui) Co., Ltd. The resulting sequences were queried against the GenBank database using BLAST to identify homologous sequences(BLAST criteria: sequence similarity ≥ 97%, query coverage ≥ 99%, E-value ≤ 1e-50). Phylogenetic trees were constructed in MEGA 7.0 using the maximum likelihood (ML) method, and branch support was assessed with 1,000 bootstrap replicates. Final taxonomic assignments were determined by integrating (i) phylogenetic clustering patterns, (ii) the closest type strains or well-characterized reference strains, and (iii) percentage sequence similarity.

Identification of endophytic bacteria: Endophytic bacterial isolates obtained from *P. praeruptorum* tissues were inoculated on LB agar plates. Single colonies were inoculated into LB broth and cultured with shaking, after which bacterial cells were harvested for DNA extraction. Total genomic DNA was extracted using a commercial bacterial genomic DNA extraction kit. The bacterial 16S rRNA gene was amplified by PCR using the universal primer pair 27F (5′-AGAGTTTGATCCTGGCTCAG-3′) and 1492R (5′-GGTTACCTTGTTACGACTT-3′) (Oleg and Foy, 2011). PCR products were verified on 1.0% (w/v) agarose gels and sequenced by General Biol (Anhui) Co., Ltd. The obtained sequences were compared with reference sequences in GenBank using BLAST, and phylogenetic trees were constructed in MEGA 7.0 using the ML method.

### 2.6 Screening for plant growth-promoting traits

Phosphate-solubilizing ability (Priya et al., 2022): For fungal isolates, mycelial plugs with a diameter of 5 mm were obtained using a sterile cork borer, whereas single bacterial colonies were picked for inoculation. The isolates were inoculated onto solid media containing either organic or inorganic phosphate (Pandey and Saharan, 2025b) and incubated at 28 °C for 3 days. The formation of clear halos surrounding the colonies indicated phosphate-solubilizing activity.

Potassium-solubilizing ability (Pandey and Saharan, 2025): Fungal mycelial plugs (5 mm in diameter) and single bacterial colonies were inoculated onto potassium-solubilizing agar media and incubated at 28 °C for 3 days. The appearance of a transparent halo around the colony was considered indicative of potassium-solubilizing activity.

After incubation, the colony diameter (D) and the diameter of the transparent hydrolysis halo (H) were measured using a vernier caliper.

The solubilization index (SI) was calculated using the following formula: SI = H/D

Where:

SI = solubilization index (unitless)

H = diameter of the transparent halo (mm)

D = diameter of the microbial colony (mm)

A higher SI value indicates stronger phosphate-solubilizing or potassium-solubilizing activity. Each strain was tested in triplicate, and the mean ± standard error (SE) was calculated.

### 2.7 Co-cultivation of functional strains with *A. thaliana* on plates

Surface sterilization of *A. thaliana* seeds: immersed in 70% ethanol for 10 min, absolute ethanol for 1 min, and 3% sodium hypochlorite for 1 min, followed by rinsing with sterile water 3–4 times. Ten milliliters of MS, PDA, and LB media were poured into dual-compartment petri dishes respectively(In order to a certain extent preventing physical contact while permitting chemical exchange). After solidification, the sterilized *A. thaliana* seeds were gently spotted on the MS medium(7–9 seeds were placed per compartment, with 3 biological replicates per treatment). Four fungal strains (G29, G30, G31, J8) with excellent plant growth−promoting traits, including organic phosphorus solubilization, inorganic phosphorus solubilization, and potassium solubilization, were selected. Three bacterial strains (PG1, PY3, PY4) were also chosen for subsequent experiments. Fungal mycelial plugs with a diameter of 5 mm were obtained from actively growing PDA plates using a sterile cork borer, ensuring consistency in mycelial mass, thickness, and viability. The plugs were then placed mycelium-side down onto the PDA region of the dual-compartment plates, with one plug per treatment. Bacterial strains were inoculated onto the LB region using the streak plate method at a density of 1×10^6^ CFU/mL. Dual-compartment petri dishes with only MS medium served as the control, and each treatment was set with 3 replicates. The Petri dishes were sealed with Parafilm to maintain humidity. The prepared petri dishes were placed in a light incubator at 25 °C under a 16 h/8 h (light/dark) photoperiod. After 21 days of cultivation, parameters including fresh weight, root length, and number of lateral roots of *A. thaliana* seedlings were measured, and the contents of chlorophyll, soluble sugars, and soluble proteins were determined.

### 2.8 Evaluation of the growth-promoting effects of plant growth-promoting strains on potted *P. praeruptorum*

After surface sterilization (as described in Section 2.1), seeds of *P. praeruptorum* were evenly spread on MS medium. The Petri dishes were incubated in a tissue culture room at 25 °C under a 16 h light/8 h dark photoperiod. The potting medium (vermiculite: perlite: sandy loam = 1: 1: 2) was sterilized and placed into plastic pots (wiped with 75% ethanol). *P. praeruptorum* seedlings at the two-leaf stage were then transplanted into the pots and cultivated at 25 °C, 70% relative humidity, and a 12 h light/12 h dark photoperiod, with three biological replicates per group. The endophytic strains that performed excellently in the *A. thaliana* co-culture assay were selected to prepare bacterial suspensions. Each *P. praeruptorum* seedling was irrigated with 2 mL of the suspension (OD_600_ = 1.0, prepared in sterile water) every 3 days, while the control group received 2 mL of sterile water. After 45 days of cultivation, the seedlings were harvested for the determination of physiological and biochemical indicators, including fresh weight, root length, plant height, chlorophyll content, soluble sugar content, soluble protein content, superoxide dismutase (SOD), peroxidase (POD), and catalase (CAT).

## 3 Results

### 3.1 Overall sequencing output and alpha diversity analysis of microbial communities

**Figure 1:**
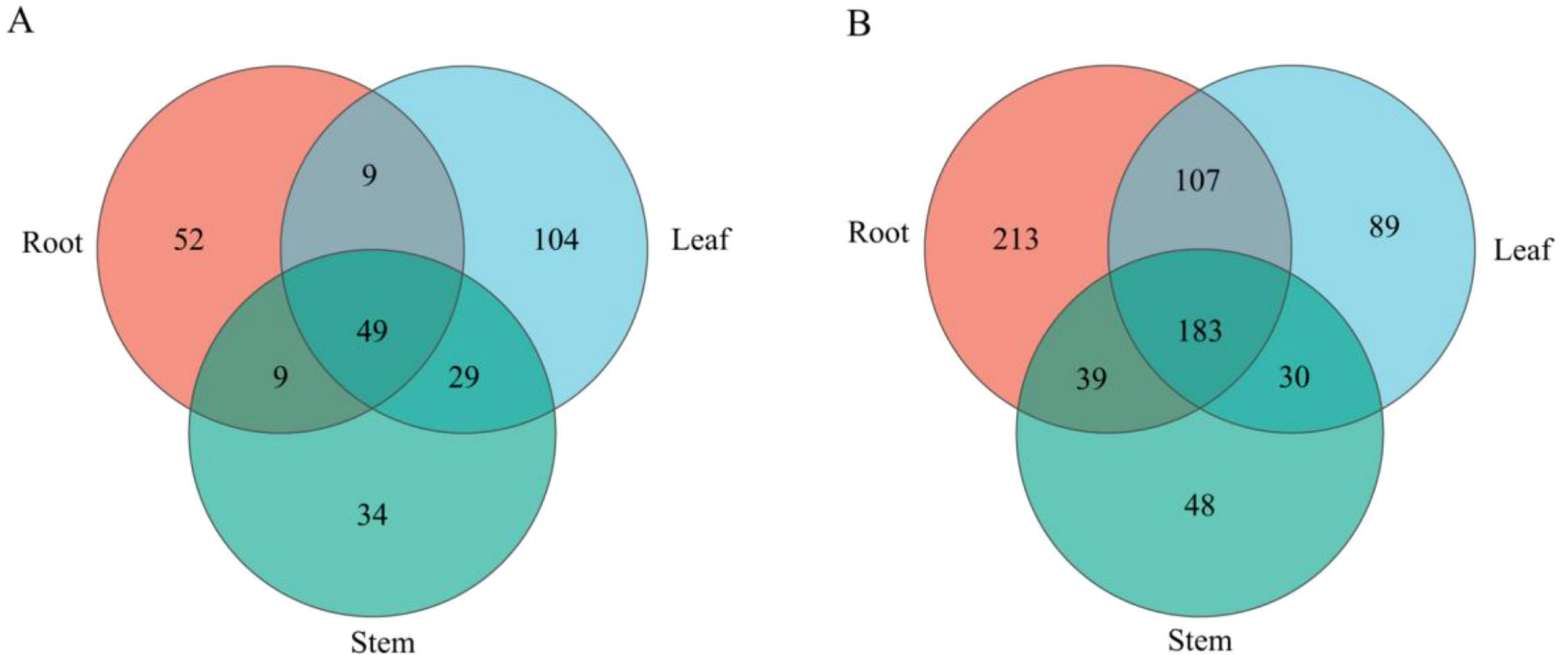
Venn diagrams of endophytic microbial communities in different tissues of *P. praeruptorum.* **(A)** Endophytic fungi; **(B)** Endophytic bacteria.

**Figure 2:**
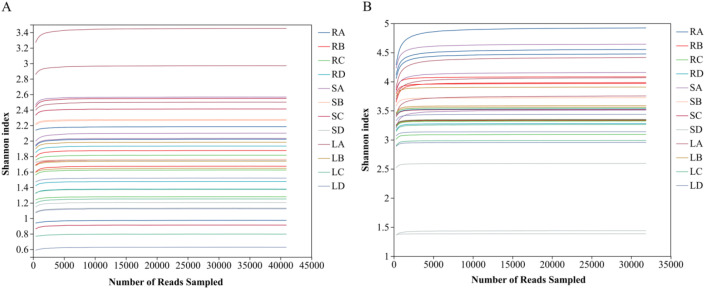
Rarefaction curves of endophytic microbial communities associated with *P. praeruptorum.* **(A)** Endophytic fungi; **(B)** Endophytic bacteria. R, S, and L represent root, stem, and leaf tissues, respectively; A, B, C, and D correspond to the samples collected in September, October, November, and December.

HTS of the ITS and 16S rRNA gene regions generated a total of 2,468,536 high-quality fungal reads and 2,250,474 high-quality bacterial reads. These sequences were clustered into 2,592 fungal ASVs and 6,705 bacterial ASVs (**Tables 2**, **3**). Based on ASV-based taxonomic annotation, Venn diagrams were used to examine the distribution of endophytic fungi and bacteria among different plant tissues (roots, stems, and leaves) (**Figure 1**). At the fungal genus level, 49 ASVs were shared among all three tissues, while the numbers of tissue-specific fungal ASVs were 52, 34, and 104 in roots, stems, and leaves. At the bacterial genus level, 183 ASVs were common to roots, stems, and leaves, while the numbers of tissue-specific bacterial ASVs were 213, 48, and 89 in roots, stems, and leaves, respectively. Rarefaction curves reached a plateau at the current sequencing depth, indicating that the sequencing effort was sufficient to capture the majority of microbial taxa present in the samples. Therefore, the obtained sequencing data reliably reflected the overall species richness and could be effectively used for subsequent analyses of endophytic microbial diversity in *P. praeruptorum* (**Figure 2**).

**Table 2:** Alpha diversity indices of endophytic fungal communities in different tissues of *P. praeruptorum* at different sampling times.

Sample	ACE	Chao	Shannon index	Simpson index
RA	73.41 ± 8.20^Aa^	73.33 ± 8.14^Aa^	1.73 ± 0.65^Aa^	0.34 ± 0.23^Cc^
RB	66.33 ± 12.66^Cc^	66.33 ± 12.66^Bb^	1.77 ± 0.10^Ba^	0.34 ± 0.04^Ca^
RC	52.67 ± 10.69^Bc^	52.67 ± 10.69^Ca^	1.57 ± 0.27^Cc^	0.39 ± 0.10^Ab^
RD	59.00 ± 16.46^Ca^	59.00 ± 16.46^Ca^	1.60 ± 0.30^Cc^	0.38 ± 0.10^Aa^
SA	97.32 ± 26.61^Ac^	98.00 ± 27.78^Ac^	2.23 ± 0.29^Ba^	0.26 ± 0.07^Bb^
SB	48.33 ± 8.33^Ba^	48.33 ± 8.33^Ba^	2.10 ± 0.30^Ba^	0.27 ± 0.08^Bb^
SC	70.77 ± 28.02^Bc^	70.17 ± 28.44^Bc^	1.96 ± 0.90^Ca^	0.37 ± 0.28^Ac^
SD	42.33 ± 16.77^Bb^	42.33 ± 16.77^Bb^	1.36 ± 0.34^Ba^	0.49 ± 0.18^Ba^
LA	200.04 ± 70.09^Ab^	197.65 ± 71.54^Ab^	2.97 ± 0.48^Ab^	0.12 ± 0.08^Cb^
LB	37.15 ± 32.25^Ca^	59.67 ± 7.64^Ca^	1.79 ± 0.17^Ab^	0.31 ± 0.02^Cb^
LC	50.67 ± 6.11^Ac^	50.67 ± 6.11^Ac^	1.14 ± 0.30^Ab^	0.51 ± 0.13^Ba^
LD	49.31 ± 2.13^Cb^	48.67 ± 2.31^Cb^	1.09 ± 0.44^Cb^	0.61 ± 0.19^Ab^

(R, S, and L represent root, stem, and leaf tissues, respectively; A, B, C, and D correspond to the samples collected in September, October, November, and December). Values are mean ± standard error (SE) (n = 3). Different capital letters A, B, and C represent the significant differences between different parts (roots, stems, and leaves) of the same time, and different lowercase letters a, b, and c represent the significant differences between different times of the same part, *p* < 0.05.

**Table 3:** Alpha diversity indices of endophytic bacterial communities in different tissues of *P. praeruptorum* at different sampling times.

Sample	ACE	Chao	Shannon index	Simpson index
RA	619.32 ± 113.71^Ab^	620.95 ± 115.15^Ab^	4.66 ± 0.23^Ac^	0.02 ± 0.00^Bc^
RB	191.07 ± 84.72^Ba^	189.84 ± 83.80^Ba^	4.07 ± 0.06^Ba^	0.04 ± 0.02^Cb^
RC	81.63 ± 15.95^Bc^	80.73 ± 15.39^Bc^	3.35 ± 0.23^Cb^	0.07 ± 0.02^Ab^
RD	96.57 ± 9.29^Cc^	96.31 ± 10.35^Cc^	3.46 ± 0.11^Cc^	0.06 ± 0.00^Ac^
SA	369.76 ± 55.04^Aa^	368.57 ± 54.95^Aa^	4.14 ± 0.57^Aa^	0.07 ± 0.07^Cc^
SB	92.70 ± 5.42^Ac^	90.98 ± 5.34^Ac^	3.55 ± 0.18^Ac^	0.05 ± 0.01^Cc^
SC	78.16 ± 12.01^Ca^	77.78 ± 12.33^Ca^	3.42 ± 0.11^Ca^	0.06 ± 0.00^Ba^
SD	63.41 ± 29.63^Bb^	62.98 ± 29.22^Bb^	1.85 ± 0.66^Bc^	0.27 ± 0.12^Bb^
LA	510.29 ± 102.80^Ac^	507.29 ± 100.98^Ac^	4.11 ± 0.34^Ab^	0.05 ± 0.01^Ac^
LB	102.62 ± 21.81^Bc^	100.96 ± 20.8^Bc^	3.62 ± 0.29^Bb^	0.05 ± 0.02^Ca^
LC	89.46 ± 13.75^Cb^	87.25 ± 11.63^Cb^	3.27 ± 0.22^Bc^	0.08 ± 0.03^Bb^
LD	73.72 ± 22.12^Cc^	74.42 ± 23.93^Cc^	3.20 ± 0.26^Cc^	0.07 ± 0.02^Aa^

(R, S, and L represent root, stem, and leaf tissues, respectively; A, B, C, and D correspond to the samples collected in September, October, November, and December). Values are mean ± standard error (SE) (n = 3). Different capital letters A, B, and C represent the significant differences between different parts (roots, stems, and leaves) of the same time, and different lowercase letters a, b, and c represent the significant differences between different times of the same part, *p* < 0.05.

Alpha diversity analysis was conducted using multiple indices to evaluate microbial community richness and diversity across samples. In this study, four alpha diversity indices, ACE, Chao, Shannon, and Simpson, were applied to comprehensively characterize the diversity of endophytic microbial communities in *P. praeruptorum* at different sampling times. The ACE and Chao indices were used to estimate species richness, the Shannon index integrates both species richness and evenness, whereas the Simpson index emphasizes the influence of dominant taxa within the community.

As shown in **Tables 2**, **3**, alpha diversity analysis revealed pronounced temporal and tissue-specific patterns. In the fungal communities, the ACE and Chao indices in September were significantly higher than those in other months, indicating the highest fungal species richness during this period. The LA group exhibited a significantly higher Shannon index and the lowest Simpson index. Combined with the ACE/Chao indices, these results indicate that the microbial community not only had higher species richness but also an extremely even species distribution (the Shannon index reflects both richness and evenness, while a low Simpson index indicates weak dominance by dominant species). In contrast, the SD, LC, and LD groups exhibited higher Simpson indices, indicating a stronger dominance of specific fungal taxa in these communities. In bacterial communities, the ACE, Chao, and Shannon indices across different tissues exhibited an overall decreasing trend over time, with significantly higher diversity indices in September than in the other months, indicating the highest bacterial richness and more even community composition during this period. The Shannon index further indicated that in September and October, fungal diversity in roots was significantly lower than in stems and leaves, whereas bacterial diversity in roots was higher than that in stems and leaves during the same period. In November, both fungal and bacterial diversity were highest in stems, followed by leaves and roots. In December, the fungal and bacterial diversity in roots was significantly higher than that in stems and leaves. Overall, these results demonstrate that the diversity of endophytic microbial communities in *P. praeruptorum* is significantly influenced by both plant organ (root, stem, and leaf) and sampling time.

### 3.2 Beta diversity analysis of endophytic fungal and bacterial communities in *P. praeruptorum*

**Figure 3:**
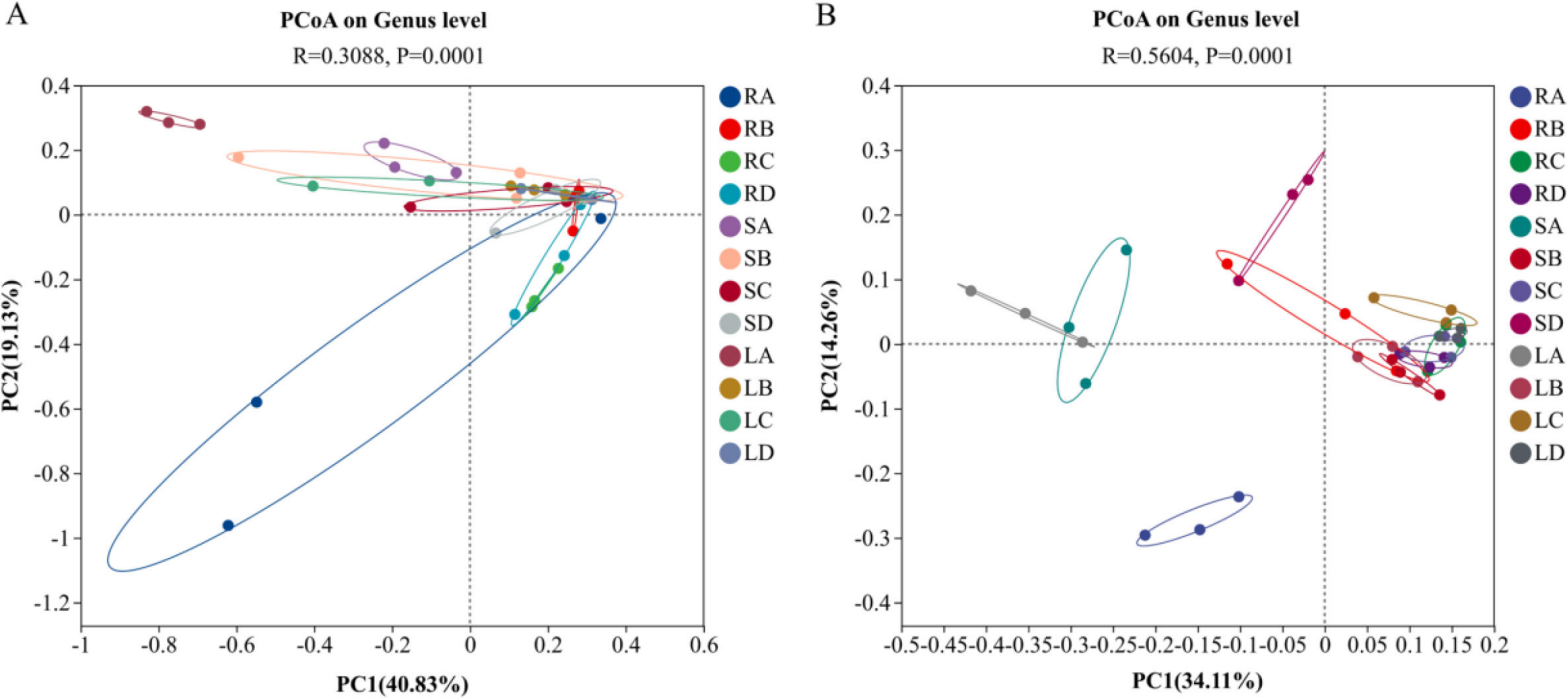
Principal coordinates analysis (PCoA) of endophytic microbial communities in *P. praeruptorum.* **(A)** Endophytic fungi; **(B)** Endophytic bacteria. R, S, and L represent root, stem, and leaf tissues, respectively; A, B, C, and D correspond to the samples collected in September, October, November, and December.

PCoA based on weighted UniFrac distances at the genus level was performed to assess the beta diversity of endophytic fungal and bacterial communities, and intergroup differences were evaluated using ANOSIM (**Figure 3**). For fungal communities (**Figure 3A**), the first principal coordinate (PC1) and the second principal coordinate (PC2) explained 40.83% and 19.13% of the total variation, respectively, together accounting for 60.0% of the community structural variation, indicating that these two axes effectively captured the differences in community composition among groups. ANOSIM results (R = 0.3088, *P* = 0.0001) further demonstrated highly significant differences in fungal community structure among different tissue–time combinations (*P* < 0.001). In the PCoA plot, samples from roots showed limited overlap with those from stems and leaves along both PC1 and PC2 axes, suggesting a pronounced separation of root-associated fungal communities from those of aboveground tissues. In contrast, stem and leaf samples were clustered in the central-right region of the ordination space with partial overlap, indicating a weaker differentiation between these two tissues compared with their differentiation from roots. For bacterial communities (**Figure 3B**), PC1 and PC2 explained 34.11% and 12.26% of the total variation, respectively, accounting for 46.37% of the overall community structural variation. ANOSIM analysis revealed a higher degree of separation among groups (R = 0.5604, *P* = 0.0001), indicating highly significant differences in bacterial community structure among different tissue–time combinations. Notably, samples collected in September were clearly separated from those collected in the other three months for roots, stems, and leaves, whereas samples from October to December exhibited substantial overlap. This pattern suggests that the bacterial community structure in September differed markedly from that in the later sampling periods.

### 3.3 Composition of fungal and bacterial communities

**Figure 4:**
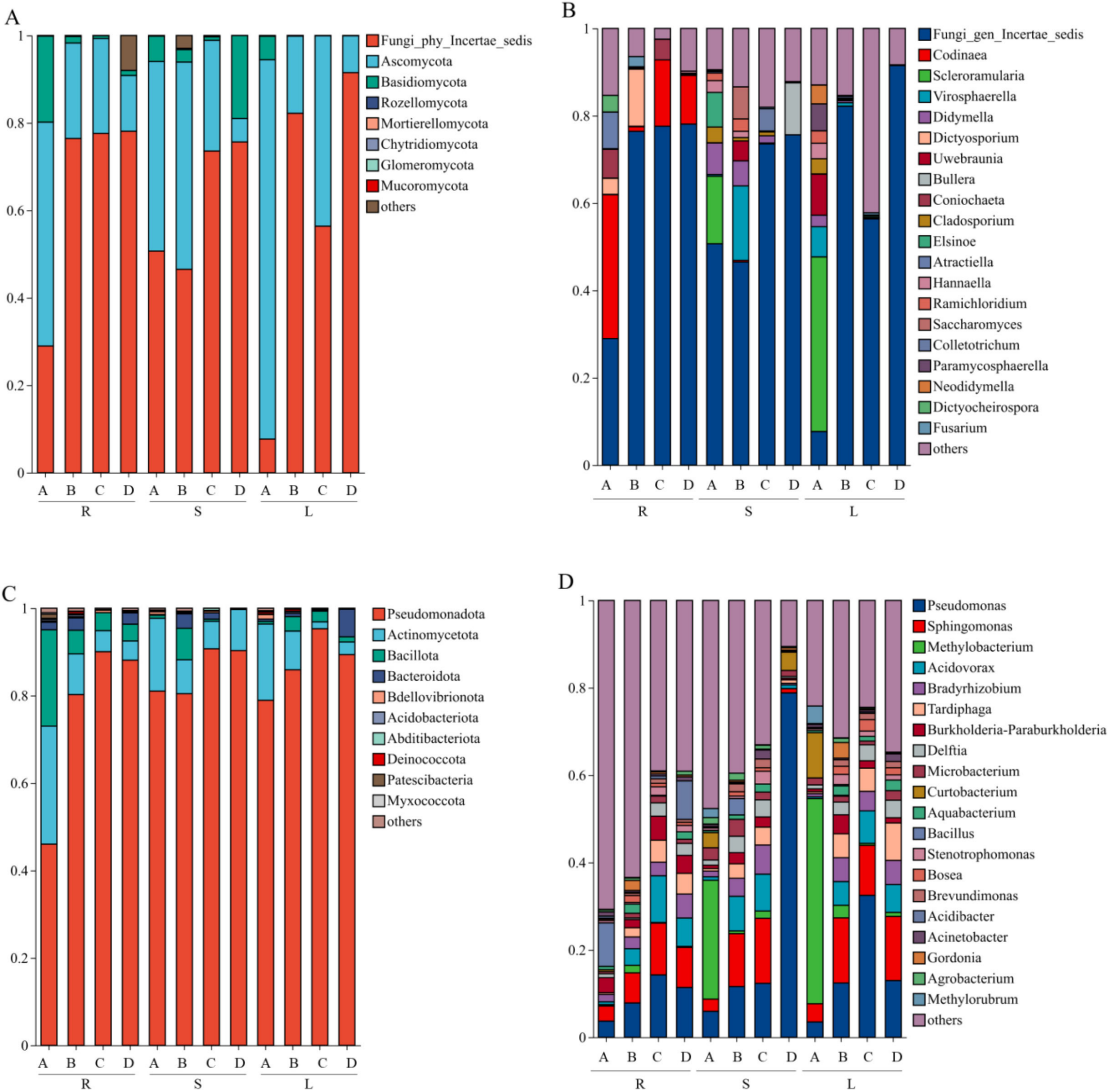
Stacked bar charts illustrating the relative abundance profiles of **(A)** fungal phyla, **(B)** fungal genera, **(C)** bacterial phyla, and **(D)** bacterial genera. The x-axis indicates sample groups categorized by tissue type and collection time; the y-axis represents the relative abundance of each taxon. R, S, and L represent root, stem, and leaf tissues, respectively; A, B, C, and D correspond to the samples collected in September, October, November, and December.

The compositions of fungal and bacterial communities associated with *P. praeruptorum* at different sampling times were analyzed (**Figure 4**). Within the fungal communities, Ascomycota and Basidiomycota were the dominant phyla (**Figure 4A**). Ascomycota predominated in nearly all samples, except for stem samples collected in December, in which Basidiomycota was the dominant phylum. At the genus level, both plant tissue type and sampling time strongly influenced fungal community composition (**Figure 4B**). Excluding unclassified genera, the dominant genus in roots was *Codinaea* in most sampling periods, whereas *Dictyosporium* dominated root samples in October. In stems, the dominant fungal genera exhibited clear temporal succession, with *Scleroramularia* in September, *Virosphaerella* in October, *Colletotrichum* in November, and *Bullera* in December. In leaves, fungal community composition was most diverse and abundant in September, with *Scleroramularia*, *Uwebraunia*, and *Paramycosphaerella* being the three most abundant genera. In contrast, during the remaining sampling periods, leaf-associated fungal communities were mainly composed of genera with relatively low abundances (relative abundance < 1%, such as *Virosphaerella and Colletotrichum*).

At the phylum level, bacterial community analysis revealed that the four most abundant phyla were Pseudomonadota, Actinomycetota, Bacillota, and Bacteroidota (**Figure 4C**). Among them, Pseudomonadota was clearly dominant, with relative abundances ranging from 45.89% to 95.15%. However, the relative abundances of dominant bacterial phyla differed among plant tissues: Pseudomonadota was slightly more abundant in stems and leaves than in roots, whereas Actinomycetota and Bacillota were relatively more abundant in roots than in aboveground tissues. At the genus level, bacterial community structures within the same tissue were generally similar across different sampling times (**Figure 4D**). The dominant bacterial genera included *Pseudomonas*, *Sphingomonas*, *Acidovorax*, *Methylobacterium*, *Bradyrhizobium*, *Tardiphaga*, *Burkholderia*, and *Delftia*. Notably, the relative abundances of some genera exhibited consistent temporal trends within the same tissue. For example, in root samples, the relative abundances of *Pseudomonas*, *Sphingomonas*, and *Acidovorax* increased initially and then decreased over time, reaching peak levels in November.

### 3.4 Functional prediction of endophytic fungal and bacterial communities in *P. praeruptorum*

**Figure 5:**
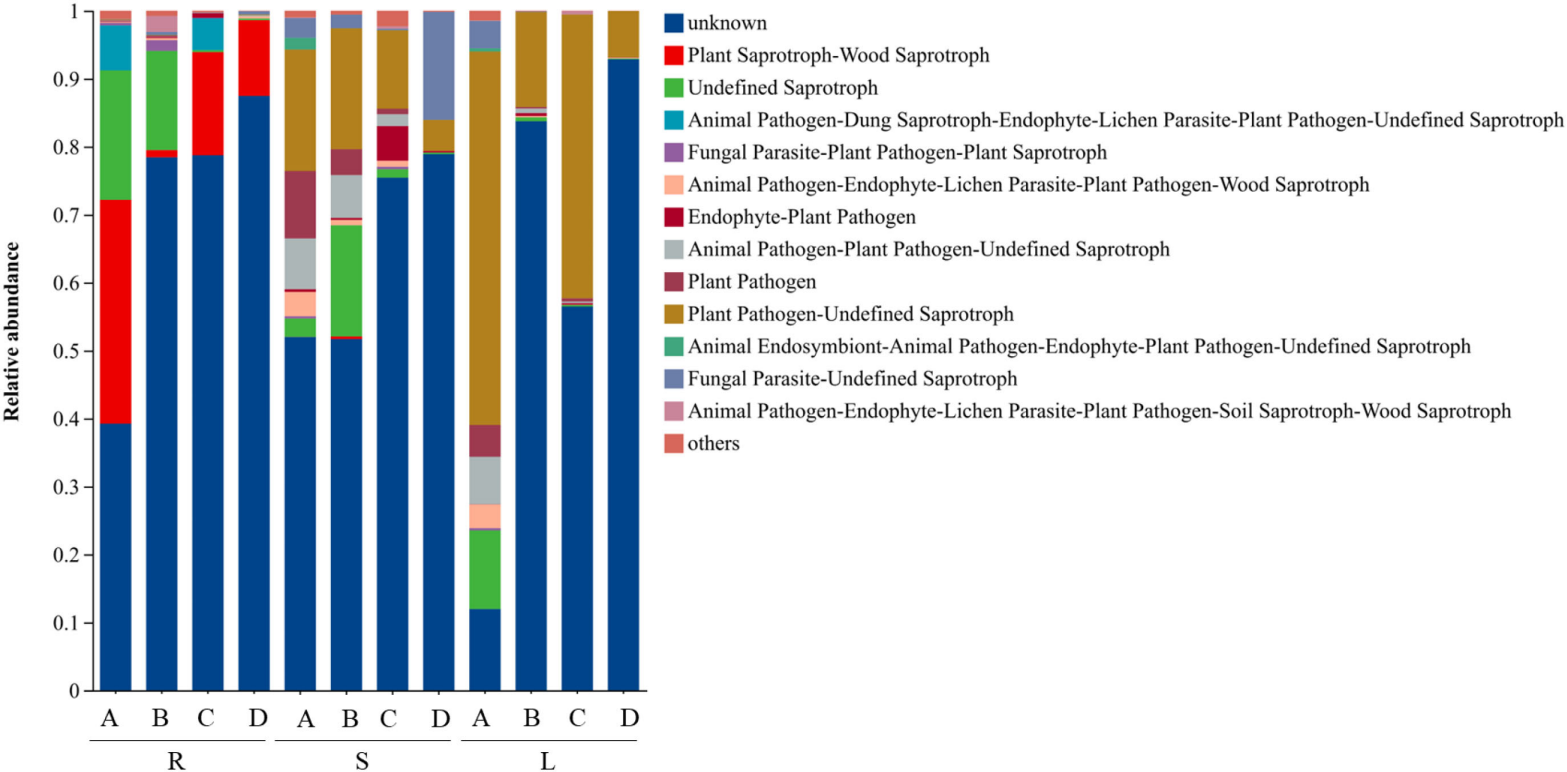
Variations in the composition of fungal functional groups inferred by FUNGuild. R, S, and L represent root, stem, and leaf tissues, respectively; A, B, C, and D correspond to the samples collected in September, October, November, and December.

Functional guilds of endophytic fungi from different tissues of *P. praeruptorum* were predicted based on the FUNGuild database (**Figure 5**). The results showed that, after excluding guilds with unknown functions, plant saprotroph-wood saprotroph was the potential ecological functional group in root-associated endophytic fungal communities. Undefined saprotrophs were widely distributed across root, stem, and leaf tissues and accounted for relatively high proportions in the RA, RB, SA, SB, and LA samples. In contrast, plant pathogen–undefined saprotrophs constituted the potential ecological functional group in endophytic fungal communities associated with stems and leaves. In addition, several functional guilds were predicted across all three tissue types, including fungal parasite-plant pathogen-plant saprotroph, plant pathogen-endophyte-lichen parasite-plant pathogen-wood saprotroph, endophyte-plant pathogen, animal pathogen-plant pathogen-undefined saprotroph, plant pathogen, and fungal parasite-undefined saprotroph.

**Figure 6:**
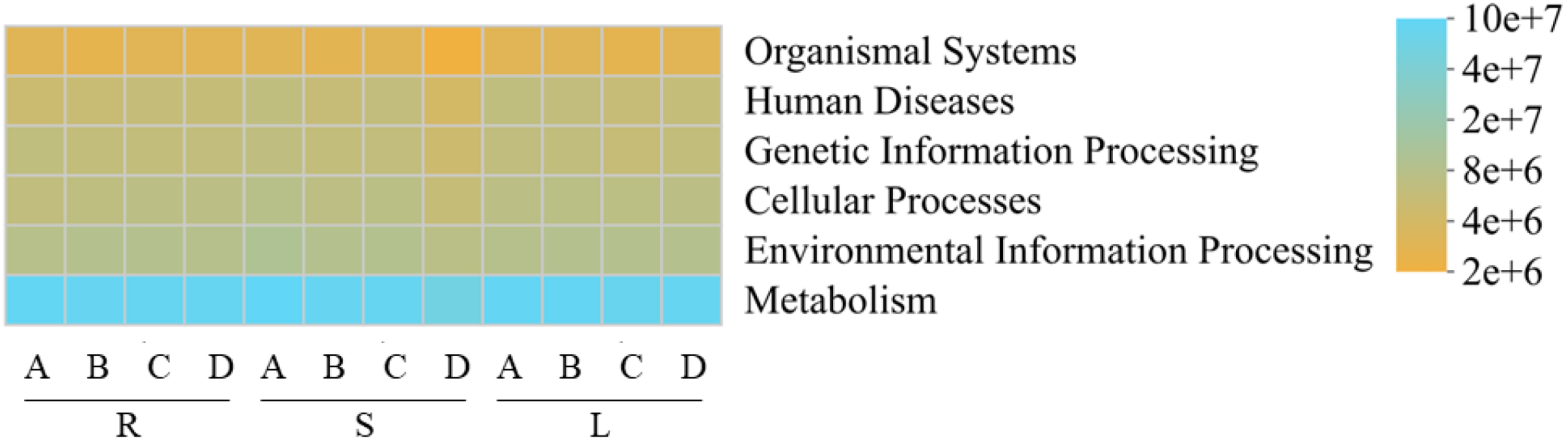
Heatmap of predicted bacterial functions based on KEGG Pathway Level 1 classification. R, S, and L represent root, stem, and leaf tissues, respectively; A, B, C, and D correspond to the samples collected in September, October, November, and December.

**Figure 7:**
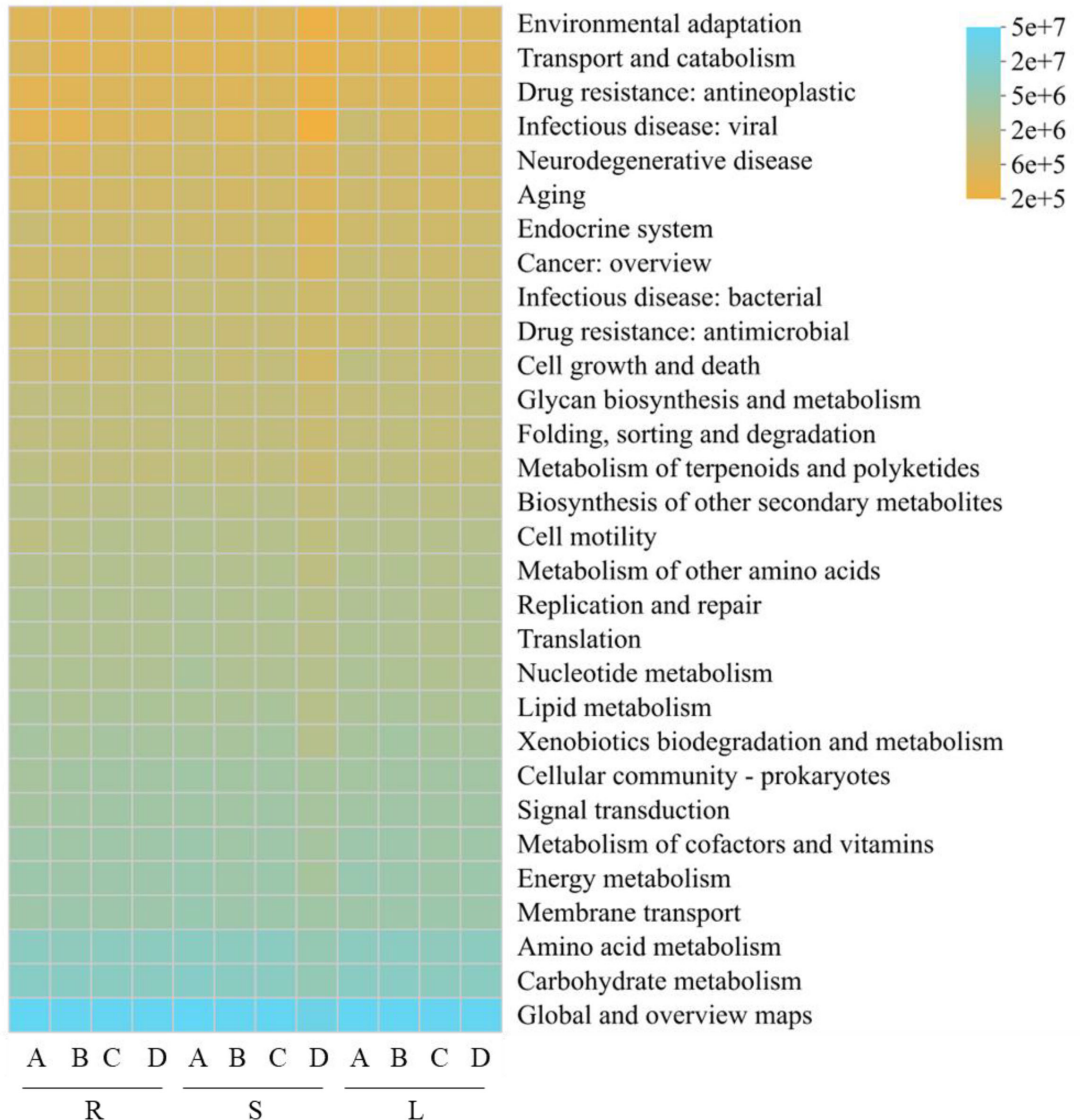
Heatmap of predicted bacterial functions based on KEGG pathway level 2 classification. R, S, and L represent root, stem, and leaf tissues, respectively; A, B, C, and D correspond to the samples collected in September, October, November, and December.

**Figure 8:**
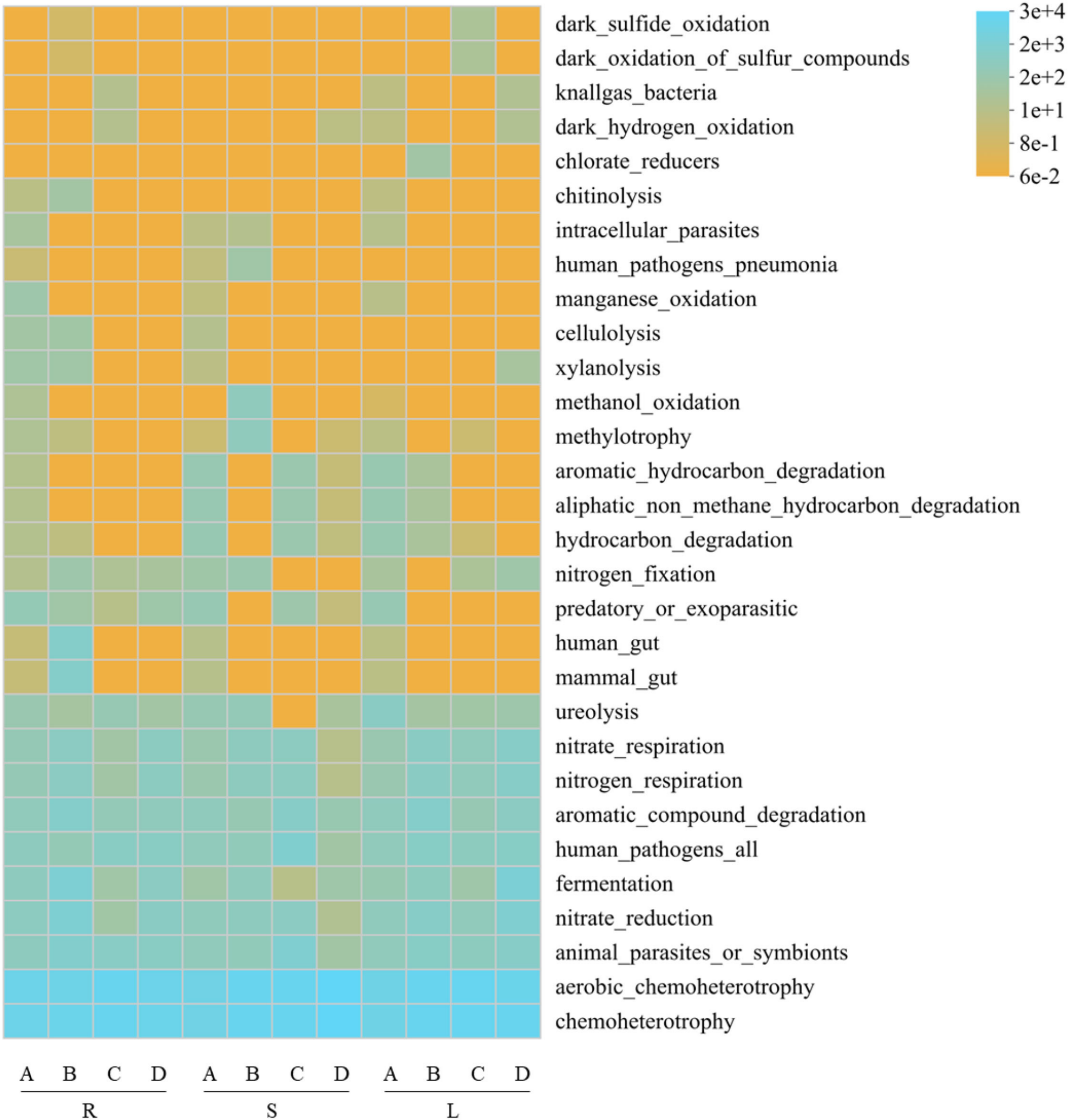
Heatmap of predicted bacterial ecological functions based on FAPROTAX annotation. R, S, and L represent root, stem, and leaf tissues, respectively; A, B, C, and D correspond to the samples collected in September, October, November, and December.

Functional prediction of bacterial communities from different tissues of *P. praeruptorum* was performed based on the PICRUSt2(mean NSTI score = 0.12 ± 0.03) and FAPROTAX databases (**Figures 6**–**8**). PICRUSt2-based prediction identified six level-1 functional categories (**Figure 6**), including metabolism, environmental information processing, cellular processes, genetic information processing, human diseases, and organismal systems. Among these, metabolism was by far the most dominant category, accounting for more than 73% of the total relative abundance. Within these six level-1 categories, a total of 46 level-2 functional pathways were annotated (**Figure 7**), of which 19 pathways exhibited relative abundances greater than 1%.

These pathways were mainly associated with global and overview maps, carbohydrate metabolism, amino acid metabolism, and other core metabolic functions, indicating that the endophytic bacterial communities of *P. praeruptorum* are primarily involved in fundamental metabolic processes.

Based on FAPROTAX functional annotation (**Figure 8**), the potential functions of endophytic bacterial communities in the roots, stems, and leaves of *P. praeruptorum* were mainly associated with chemoheterotrophy, aerobic chemoheterotrophy, animal_parasites_or_symbionts, nitrate_reduction, fermentation, human_pathogens_all, aromatic_compound_degradation, nitrogen_respiration, and nitrate_respiration. Microbial fermentation (14.47%) represented the potential metabolic process of these endophytic bacterial communities, followed by nitrate reduction (5.08%), nitrate respiration (1.78%), nitrogen respiration (1.78%), and nitrogen fixation (2.30%), it is predicted that this endophytic bacterial community may be involved in the nitrogen biogeochemical cycle. In terms of trophic modes, chemoheterotrophic bacteria were the most abundant (29.70%), whereas photoautotrophic (0.22%) and photoheterotrophic (0.29%) bacteria were present at relatively low abundances. In addition, potential pathogenic taxa annotated by FAPROTAX were also detected, including animal parasites (5.33%), human gut microbiota (3.50%), mammalian gut microbiota (3.50%), and human pathogens (3.13%), with a total relative abundance of 15.61%.

### 3.5 Isolation and identification of endophytic microorganisms in *P. praeruptorum*

Using tissue isolation and plate streaking methods, a total of 212 culturable endophytic microbial strains were isolated from the roots, stems, and leaves of *P. praeruptorum*, including 173 endophytic fungal strains and 39 endophytic bacterial strains. The isolation rates of endophytic fungi from roots, stems, and leaves were 61%, 57%, and 55%, respectively, indicating that all tissues harbor abundant culturable fungal resources. All isolates were identified by sequencing of the fungal ITS rRNA region (**Supplementary Table S1**) and the bacterial 16S rRNA gene (**Supplementary Table S2**). The results showed that over 90% of the sequences shared ≥ 97% similarity with the type strains, a threshold suitable for identification at the genus level; thus, species-level identification was considered tentative. The phylogenetic tree constructed using the maximum-likelihood method supported the taxonomic assignment at the genus level, whereas some species-level clades showed low bootstrap support (< 50%) with limited resolution (**Supplementary Figures S1**–**S4**). The 173 endophytic fungal isolates were assigned to 31 genera, with a clear dominance pattern in genus-level composition. *Aspergillus*, *Arcopilus*, and *Colletotrichum* were identified as the core dominant genus among the culturable strains, comprising 56, 22, and 18 isolates, respectively, and together accounting for 55.49% of the total fungal isolates. *Didymella* and *Penicillium* were also common genera, with 13 and 13 isolates, respectively, collectively representing 15.03% of the total. The remaining genera were classified as rare genera and together accounting for 29.48% of the total, reflecting the high diversity of endophytic fungi associated with *P. praeruptorum*. The 39 endophytic bacterial isolates belonged to 16 genera and also exhibited a dominance-driven community structure. *Curtobacterium*, *Pseudomonas*, *Bacillus*, and *Paenibacillus* were the dominant genus among the culturable bacterial strains, represented by 9, 6, 4, and 4 isolates, respectively, together accounting for 58.97% of the total bacterial isolates.

### 3.6 PGP traits of endophytic microorganisms

To evaluate the PGP potential of the isolated strains, *in vitro* functional assays were conducted on the purified endophytic fungal and bacterial isolates. The phosphate-solubilizing and potassium-solubilizing activities of the strains were quantitatively evaluated based on the solubilization index (SI). A higher SI value indicates stronger functional activity (**Supplementary Table S3**; **Supplementary Figure S5**). The phosphate-solubilization assays showed that among the 173 endophytic fungal isolates, 52 strains produced clear halos on organic phosphate medium, while 29 strains formed clear halos on inorganic phosphate medium. Among the 39 endophytic bacterial isolates, 8 strains exhibited organic phosphate–solubilizing activity, and 3 strains were capable of solubilizing inorganic phosphate. Isolates showed substantial variation in phosphate-solubilizing activity among isolates. Strain J53 exhibited the strongest organic phosphate-solubilizing ability, with a SI value of 2.64 ± 0.21, whereas strain PY4 showed the highest inorganic phosphate-solubilizing activity, with a SI value of 1.46 ± 0.16. Overall, the tested isolates displayed stronger organic phosphate-solubilizing activity than inorganic phosphate-solubilizing activity. Potassium-solubilization screening revealed that 37 of the 173 endophytic fungal isolates formed clear halos on potassium-solubilizing medium, indicating potassium-solubilizing activity. Marked differences in potassium-solubilizing capacity were detected among strains. Strain J8 exhibited the highest potassium-solubilizing activity, with a SI value of 2.00 ± 0.38, significantly outperforming other isolates. Strains G14 and G16 also demonstrated strong potassium-solubilizing potential, with SI values of 1.92 ± 0.20 and 1.83 ± 0.09, respectively. Potassium-solubilization screening revealed that 3 of the 39 endophytic bacterial isolates formed clear halos on potassium-solubilizing medium. The potassium-solubilizing ability of strain PY3 was the strongest among all the tested strains, reaching 2.48 ± 0.19.

### 3.7 Plant growth-promoting effects of endophytic strains on *A. thaliana*

*Arabidopsis thaliana* was co-cultured with the identified endophytic strains in dual-compartment Petri dishes for 21 days (**Table 4**). Regarding lateral root formation, inoculation with *Aspergillus foetidus* (G30) and *Talaromyces annesophieae* (G31) exerted the most significant inductive effects. *Aspergillus welwitschiae* (G29), *A. foetidus* (G30) and *T. annesophieae* (G31) markedly increased the number of lateral roots. For plant fresh weight, *A. welwitschiae* (G29), *T. annesophieae* (G31) and *Paenibacillus maysiensis* (PG1) significantly improved biomass accumulation, whereas *A. welwitschiae* (J8), *Cellulomonas pakistanensis* (PY3) and *Pantoea endophytica* (PY4) showed no significant differences compared with the non-inoculated control (CK). In terms of primary root elongation, *A. welwitschiae* (G29), *T. annesophieae* (G31) and *A. welwitschiae* (J8) obviously promoted root growth; in contrast, *P. endophytica* (PY4) significantly reduced primary root length relative to CK. Chlorophyll content was distinctly enhanced by *A. welwitschiae* (G29), *A. foetidus* (G30), *T. annesophieae* (G31) and *P. maysiensis* (PG1). Among all treatments, *T. annesophieae* (G31) achieved the highest increase in chlorophyll content (12.66%), indicating an optimal promoting effect on photosynthetic potential. Soluble sugar content was significantly elevated by *A. foetidus* (G30) and *A. welwitschiae* (J8), while most other identified endophytic strains decreased soluble sugar accumulation. The content of soluble protein was increased to varying degrees by most strains, with *A. welwitschiae* (G29) showing the strongest promotional effect; nevertheless, *P. endophytica* (PY4) significantly reduced soluble protein levels compared with the control group.

**Table 4:** The effects of different endophytic strain treatments on related indices in *A.thaliana*.

Group	Number of lateral roots	Fresh weight (mg)	Root length (cm)	Chlorophyll content (mg/g)	Soluble sugar content (mg/g)	Soluble protein content (mg/g)
CK	2.67 ± 0.58 ^a^	1.65 ± 0.40 ^a^	0.57 ± 0.03 ^a^	1.58 ± 0.01 ^a^	2.79 ± 0.02 ^a^	10.33 ± 0.08 ^a^
G29	4.33 ± 0.58 ^b^	2.89 ± 0.45 ^b^	2.52 ± 0.63 ^b^	1.76 ± 0.01 ^b^	2.74 ± 0.01 ^b^	13.65 ± 0.17 ^b^
G30	5.67 ± 0.58 ^b^	2.63 ± 0.48 ^a^	1.68 ± 0.78 ^a^	1.71 ± 0.00 ^b^	2.95 ± 0.01 ^b^	12.86 ± 0.11 ^b^
G31	5.67 ± 1.15 ^b^	3.45 ± 0.53 ^b^	2.79 ± 0.92 ^a^	1.78 ± 0.01 ^b^	2.73 ± 0.04 ^a^	11.89 ± 0.06 ^b^
J8	2.67 ± 0.58 ^a^	2.02 ± 0.20 ^a^	2.06 ± 0.73 ^a^	1.52 ± 0.01 ^b^	2.96 ± 0.02 ^b^	10.65 ± 0.03 ^b^
PG1	2.33 ± 0.58 ^a^	3.12 ± 0.13 ^b^	0.59 ± 0.02 ^a^	1.65 ± 0.01 ^b^	2.65 ± 0.02 ^b^	11.89 ± 0.10 ^b^
PY3	2.67 ± 0.58 ^a^	1.69 ± 0.12 ^a^	0.49 ± 0.01 ^b^	1.54 ± 0.01 ^b^	2.77 ± 0.02 ^a^	10.70 ± 0.06 ^b^
PY4	2.33 ± 0.58 ^a^	1.79 ± 0.12 ^a^	0.52 ± 0.02 ^a^	1.53 ± 0.01 ^b^	2.71 ± 0.04 ^a^	9.43 ± 0.08 ^b^

CK: non-microbial inoculation. All data are presented as means ± standard error (n=3). Significant differences between each treatment and CK were analyzed using Welch’s t-test. Different lowercase letters (a, b) indicate significant differences at *P* < 0.05.

### 3.8 Plant growth-promoting effects of endophytic strains on *P. praeruptorum*

**Figure 9:**
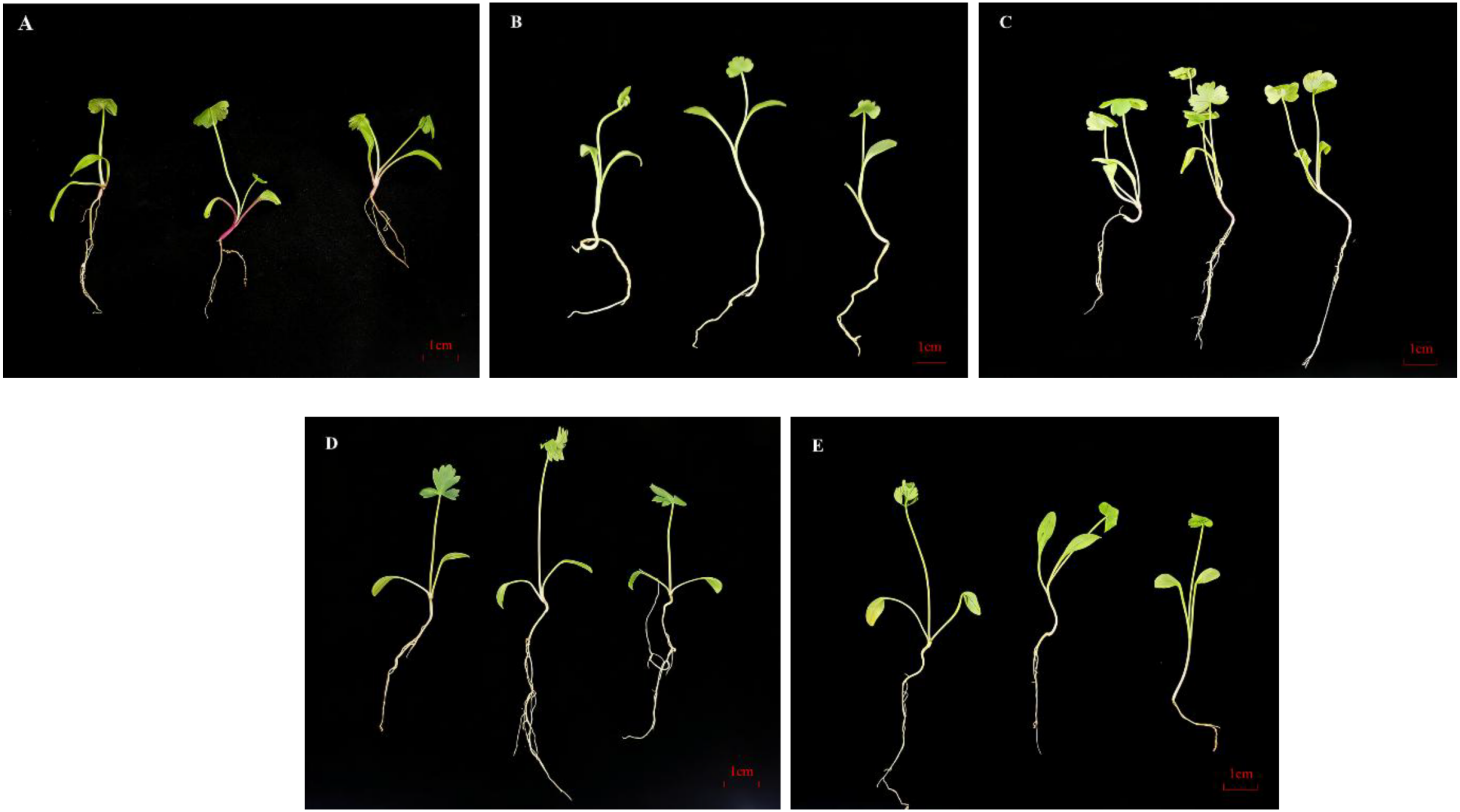
Effects of different treatments on the growth of *P. praeruptorum* after 45 days. **(A)** CK, non-microbial inoculation; **(B)** G30, *A. foetidus*; **(C)** G31, *T. annesophieae*; **(D)** G29, *A. welwitschiae*; **(E)** PG1, *P. maysiensis*.

The growth indicators (fresh weight, root length, lateral root number) of *P. praeruptorum* inoculated with identified endophytic strains are summarized in **Table 5**. Compared with the non-inoculated control (CK), inoculation with *A. welwitschiae* (G29), *A. foetidus* (G30), *T. annesophieae* (G31), and *P. maysiensis* (PG1) exerted differential regulatory effects on the growth of *P. praeruptorum*. In terms of lateral root development, *A. foetidus* and *T. annesophieae* significantly increased the number of lateral roots relative to CK, while *A. welwitschiae* showed an increasing trend and *P. maysiensis* exerted no promotional effect. For plant fresh weight, only inoculation with *A. welwitschiae* significantly enhanced the fresh biomass of *P. praeruptorum*. By contrast, *A. foetidus*, *T. annesophieae* and *P. maysiensis* improved fresh weight to a certain extent but showed no statistically significant differences compared with the control group. Regarding taproot length, all three fungal strains (*A. welwitschiae*, *A. foetidus*, *T. annesophieae*) and the bacterial strain *P. maysiensis* promoted taproot elongation at the phenotypic level; however, no significant differences were detected among all treatments according to statistical analysis (**Figure 9**).

**Table 5:** Effects of different treatments on growth indicators of *P. praeruptorum.*.

Group	Number of lateral roots	Fresh weight (mg)	Root length (cm)
CK	29.00 ± 4.24 ^a^	2.78 ± 0.97 ^a^	2.60 ± 0.46 ^a^
G29	36.34 ± 4.09 ^a^	5.11 ± 1.04 ^b^	3.27 ± 0.91 ^a^
G30	41.57 ± 4.34 ^b^	4.32 ± 0.64 ^a^	3.08 ± 0.20 ^a^
G31	40.63 ± 3.57 ^b^	4.25 ± 0.18 ^a^	3.25 ± 0.39 ^a^
PG1	27.45 ± 2.19 ^a^	3.28 ± 1.31 ^a^	3.59 ± 0.59 ^a^

CK, non-microbial inoculation. All data are presented as means ± standard error (n=3). Significant differences between each treatment and CK were analyzed using Welch’s t-test. Different lowercase letters (a, b) indicate significant differences at *P* < 0.05.

As shown in **Table 6**, inoculation with the identified endophytic strains significantly altered the physiological and biochemical characteristics of *P. praeruptorum*. Compared with the non-inoculated control (CK), inoculation with *A. welwitschiae* (G29), *A. foetidus* (G30) and *T. annesophieae* (G31) markedly increased chlorophyll content; among them, *A. welwitschiae* (G29) and *A. foetidus* (G30) exhibited extremely significant promoting effects. In addition, *P. maysiensis* (PG1) also significantly enhanced chlorophyll accumulation in *P. praeruptorum*. Regarding soluble sugar content, *A. foetidus* (G30) significantly elevated soluble sugar levels, while *P. maysiensis* (PG1) induced a significant reduction. No obvious differences in soluble sugar content were observed for *A. welwitschiae* (G29) and *T. annesophieae* (G31) relative to CK. For soluble protein accumulation, *A. welwitschiae* (G29) and *A. foetidus* (G30) achieved an extremely significant increase, with *A. welwitschiae* (G29) showing the strongest regulatory effect. By contrast, *T. annesophieae* (G31) and *P. maysiensis* (PG1) exerted no substantial influence on soluble protein content. In terms of antioxidant enzyme activity, all four strains (*A. welwitschiae*, *A. foetidus*, *T. annesophieae* and *P. maysiensis*) enhanced SOD activity. POD activity was enhanced in all inoculation groups: *A. welwitschiae* (G29) showed extremely significant upregulation, whereas *A. foetidus* (G30), *T. annesophieae* (G31) and *P. maysiensis* (PG1) presented a significant increase. For CAT activity, *A. foetidus* (G30) significantly promoted CAT activity; conversely, *T. annesophieae* (G31) and *P. maysiensis* (PG1) slightly reduced CAT activity.

**Table 6:** Effects of different treatments on physiological and biochemical indices of *P. praeruptorum*.

Group	Chlorophyll content (mg/g)	Soluble sugar content (mg/g)	Soluble protein content (mg/g)	SOD activity (U/g)	POD activity(U/g)	CAT activity (U/g)
CK	1.59 ± 0.02^a^	2.77 ± 0.02^a^	10.86 ± 0.10^a^	140.28 ± 0.86^a^	1057.25 ± 31.23^a^	274.24 ± 9.54^a^
G29	1.85 ± 0.01^b^	2.83 ± 0.01^a^	13.82 ± 0.22^b^	152.07 ± 9.50^a^	1433.88 ± 47.73^b^	280.13 ± 26.11^a^
G30	1.74 ± 0.01^b^	2.95 ± 0.09^b^	13.50 ± 0.22^b^	161.7 ± 1.33^b^	1319.37 ± 77.35^b^	309.45 ± 29.85^a^
G31	1.80 ± 0.01^b^	2.82 ± 0.04^a^	11.22 ± 0.12^b^	150.31 ± 2.35^b^	1438.03 ± 107.21^b^	267.77 ± 6.96^a^
PG1	1.64 ± 0.01^b^	2.70 ± 0.01^b^	11.06 ± 0.12^a^	148.24 ± 0.64^b^	1253.27 ± 73.33^b^	251.20 ± 4.26^b^

CK, non-microbial inoculation. All data are presented as means ± standard error (n=3). Significant differences between each treatment and CK were analyzed using Welch’s t-test. Different lowercase letters (a, b) indicate significant differences at *P* < 0.05.

## 4 Discussion

The present study revealed pronounced temporal variation in the diversity of endophytic microbial communities associated with *P. praeruptorum*. Both bacterial and fungal species richness, as indicated by the ACE and Chao indices, was significantly higher in September than in October through-December, and the bacterial community structure in September was clearly separated from those of the other sampling periods. These findings were consistent with the seasonal dynamics reported for endophytic microorganisms in many medicinal plants. For example, Lü Lixin (Lv et al., 2014) reported that endophytic fungal diversity in *Atractylodes* spp. was higher in summer than in spring and autumn, suggesting that warmer seasons generally support higher endophytic microbial diversity. In the present study, endophytic microbial diversity showed an overall declining trend over time, which may be attributed to decreasing temperatures from autumn to winter. This pattern is in agreement with observations in the alpine plant *Anisodus tanguticus*, in which reduced temperatures were associated with a decline in endophytic microbial diversity (Wang et al., 2023).

Venn diagram analysis preliminarily indicated that bacterial communities were more diverse than fungal communities. Compared with fungi, bacteria exhibited a greater number of ASVs across different plant tissues, which may be attributed to their more efficient utilization of nutrients and their high reproductive rates, allowing rapid accumulation of mutations and accelerated ASV diversification (Nemergut et al., 2013; Li et al., 2021). PCoA results further demonstrated a clear separation of endophytic microbial communities between roots and aboveground tissues (stems and leaves), as well as discernible differences between stem- and leaf-associated communities. Both plant tissue type and sampling period significantly influenced the distribution and clustering of endophytic fungal and bacterial communities in *P. praeruptorum*, indicating that endophyte colonization is dynamically shaped by ecological niche differentiation and temporal variation (Cui et al., 2022; Kentjens et al., 2025; Yan et al., 2025).

At the phylum level, the endophytic fungal community of *P. praeruptorum* exhibited a pronounced dominance pattern, with Ascomycota and Basidiomycota constituting the core components. Except for stem samples collected in December, in which Basidiomycota was dominant, Ascomycota overwhelmingly predominated in root, stem, and leaf tissues across all other sampling periods. This dominance may be attributed to the relatively rapid evolutionary rate and high species richness of Ascomycota, which confer strong environmental adaptability (Zhao et al., 2020). The endophytic bacterial community was mainly composed of Pseudomonadota, Actinomycetota, and Bacillota, with Pseudomonadota showing particularly high relative abundance (45.89%-95.15%) across different tissues and sampling times. Numerous studies have reported Pseudomonadota as a dominant bacterial phylum in plant tissues, which is consistent with our findings (Sun et al., 2017; David et al., 2021). At the genus level, *Codinaea* dominated the fungal communities in roots, while dominant genera in stems and leaves shifted with temporal changes, consistent with reports that *Codinaea* is a dominant endophytic fungal genus in *Sophora tonkinensis* (Bu et al., 2025). In bacterial communities, genera such as *Pseudomonas* and *Sphingomonas* were widely distributed. *Pseudomonas* species are well known for their PGP properties, including phytohormone (e.g., indole-3-acetic acid) production, antagonism against plant pathogens, and phosphate and potassium solubilization, thereby enhancing plant nutrient uptake and growth (Bashan et al., 2014; Lv et al., 2025; Martínez et al., 2025). *Sphingomonas* species have also been reported to possess PGP traits, such as gibberellin (GA) production and potential roles in plant disease resistance (Gong et al., 2016; Latif et al., 2016; Bilal et al., 2018).

Functional prediction analysis indicated that the endophytic fungal communities of *P. praeruptorum* were potential composed of plant saprotroph-wood saprotroph, undefined saprotroph, and plant pathogen-undefined saprotroph functional guilds. Saprotrophic fungi play crucial roles in the transformation of plant organic matter by facilitating the release and cycling of nutrients such as carbon and nitrogen, thereby directly supporting host plant growth (Nguyen et al., 2016). Functional prediction of endophytic bacterial communities based on PICRUSt2 and FAPROTAX revealed that their core functions were predominantly associated with metabolic activities. PICRUSt2 analysis showed that metabolic pathways were overwhelmingly dominant, particularly those related to carbohydrate and amino acid metabolism, suggesting that bacterial communities actively participate in the primary metabolic processes of the host plant (Noah et al., 2012). FAPROTAX annotation further indicated that chemoheterotrophy was the main trophic mode, while the detection of potential functions such as nitrate reduction, nitrogen respiration, and nitrogen fixation implies that these bacterial communities may play beneficial roles in nitrogen transformation and supply in *P. praeruptorum* (Averina et al., 2014; Xie et al., 2025). such tools only infer potential functions based on taxonomic information, and cannot directly reflect the abundance or expression levels of functional genes. The 15.61% of potential pathogenicity-related bacterial taxa annotated by FAPROTAX only represent taxonomic association predictions, and their pathogenicity has not been experimentally verified. Considering the medicinal value of *P. praeruptorum*, biosafety tests are required to eliminate potential risks during the subsequent development of microbial inoculants.

High-throughput sequencing identified Pseudomonadota and Ascomycota as the dominant taxa, while culture-dependent approaches enriched fast-growing genera such as *Aspergillus* and *Curtobacterium.* The differences resulted from medium selectivity and unculturable strains, demonstrating the complementarity of the two methods. The 173 culturable endophytic fungal isolates belonged mainly to Ascomycota, Basidiomycota, and Mucoromycota, with *Aspergillus, Arcopilus, and Colletotrichum* identified as the core dominant genus among the culturable strains, collectively accounting for 55.49% of the total isolates. These genera are typical representatives of Ascomycota, corroborating the dominant status of this phylum as revealed by sequencing analysis. Similarly, all 39 culturable endophytic bacterial isolates fell within the dominant phyla detected by HTS. *Curtobacterium*, *Pseudomonas*, *Bacillus*, and *Paenibacillus* were the core dominant genus among the culturable strains, collectively representing 58.97% of the total bacterial isolates, which is consistent with the high relative abundances of Pseudomonadota and Bacillota observed in the sequencing data. Previous studies have demonstrated that genera such as *Aspergillus*, *Arcopilus*, *Pseudomonas*, and *Bacillus* possess PGP properties or the ability to produce bioactive compounds in various medicinal plants, providing a rich resource base for subsequent development of endophytic microbial applications (Yuan et al., 2022; Sofija et al., 2023; González et al., 2025). HTS enables comprehensive coverage of both culturable and unculturable components of endophytic microbial communities and provides a detailed depiction of their spatiotemporal heterogeneity. In contrast, traditional isolation and cultivation approaches allow the acquisition of microbial strains with potential practical value, thereby supplying essential materials for subsequent functional validation and microbial inoculant development. The integration of these two approaches overcomes the limitations inherent to any single method and establishes a complete research framework, spanning from community structure characterization to the targeted mining of functionally important microbial strains.

Further screening for plant growth-promoting traits confirmed that 52 endophytic fungal isolates and 8 endophytic bacterial isolates possessed organic phosphate-solubilizing ability, while 29 fungal isolates and 3 bacterial isolates were capable of solubilizing inorganic phosphate. In addition, 37 fungal and 3 bacterial isolates exhibited potassium-solubilizing activity. Phosphorus is an essential macronutrient for plant growth and development; however, in soils it predominantly exists in insoluble organic or inorganic forms that are not readily available for direct plant uptake (Gradwell et al., 2025). Phosphate-solubilizing microorganisms can convert these insoluble phosphorus forms into bioavailable forms, thereby alleviating phosphorus fixation and improving phosphorus use efficiency, and are therefore considered key biological agents in sustainable nutrient management (Sharma et al., 2013). The results of organic phosphorus and inorganic phosphorus solubilization are consistent with previous reports and further demonstrate that endophytic fungi and bacteria represent important functional reservoirs for phosphate solubilization (Tian et al., 2016). Notably, the mechanisms underlying organic and inorganic phosphate solubilization often differ. Organic phosphate solubilization primarily relies on enzymatic systems such as phosphatases, whereas inorganic phosphate solubilization is mainly associated with the secretion of organic acids (Alori et al., 2017). Strains capable of solubilizing both organic and inorganic phosphorus may therefore activate different phosphorus pools in soil through synergistic mechanisms, enabling more efficient phosphorus mobilization. The specific metabolic pathways involved in these processes warrant further investigation. Potassium plays essential roles in plant physiological processes such as photosynthesis, enzyme activation, and assimilate transport. However, large amounts of potassium in soils are present in fixed mineral forms that cannot be directly absorbed by plants. Microorganisms can mobilize insoluble potassium through mechanisms such as organic acid production and polysaccharide secretion, thereby converting unavailable potassium into plant-accessible forms and promoting plant growth and yield (Meena et al., 2014). The potassium-solubilizing endophytic fungi screened in this study provide a valuable microbial resource for alleviating potassium limitation in agricultural systems, particularly for improving soil fertility in potassium-deficient soils. This study focused on screening core PGP traits such as phosphate solubilization and potassium solubilization, and has not verified other PGP traits including nitrate reduction and auxin content, which represented one limitation of this study. Future studies will supplement experiments on other PGP traits to further reveal the growth-promoting mechanisms of the strains.

Endophytic strains with strong plant growth-promoting traits can effectively regulate root development, enhance photosynthesis, and improve stress resistance, thus promoting host growth and nutrient accumulation (Manoj et al., 2021). Based on six physiological and growth indicators, this study investigated the comprehensive plant growth-promoting effects of seven tested strains on *A. thaliana*. *A. welwitschiae* (J8), *C. pakistanensis* (PY3) and *P. endophytica* (PY4) only slightly affected 1–2 indices and showed no overall significant effects. In contrast, *A. welwitschiae* (G29), *A. foetidus* (G30), *T. annesophieae* (G31) and *P. maysiensis* (PG1) displayed stable and efficient growth-promoting effects. *A. welwitschiae* (G29) significantly increased lateral root number, primary root length, chlorophyll content, and soluble protein content. *A. foetidus* (G30) showed strong effects on lateral root formation, fresh weight, and chlorophyll synthesis. *T. annesophieae* (G31) performed best in fresh weight, root elongation, and protein accumulation. *P. maysiensis* (PG1) significantly improved fresh weight, chlorophyll, and soluble protein contents, with strong biomass-promoting activity.

The most effective strains *A. welwitschiae* (G29), *A. foetidus* (G30), *T. annesophieae* (G31) and *P. maysiensis* (PG1) were further used for inoculation tests on *P. praeruptorum*. All four strains promoted the growth of *P. praeruptorum* to different extents. *T. annesophieae* (G31) significantly increased fresh weight, and *A. welwitschiae* (G29), *A. foetidus* (G30), and *T. annesophieae* (G31) significantly promoted taproot elongation, which is crucial for improving the yield of the medicinal root. Meanwhile, PG1, *A. welwitschiae* (G29), and G31 showed more obvious effects on plant height. These results demonstrated the stable growth-promoting effects of these strains on the host medicinal plant.

Physiological and biochemical analyses revealed that inoculation with endophytic strains significantly enhanced chlorophyll content, osmotic adjustment substances, and antioxidant enzyme activities in *P. praeruptorum*. *A. welwitschiae* (G29) and *A. foetidus* (G30) extremely significantly increased soluble protein content. All strains improved SOD and POD activities, which contributed to scavenging reactive oxygen species and enhancing stress resistance. These findings suggested that the growth-promoting mechanisms of these endophytes were related to improving photosynthesis, enhancing nutrient accumulation, and boosting antioxidant capacity (Omer et al., 2022; Zafar et al., 2022).

## 5 Conclusions

This study systematically revealed the spatiotemporal dynamics of culturable endophytic communities in *P. praeruptorum*, and obtained strains with multiple plant growth−promoting traits, including organic phosphate solubilization, inorganic phosphate solubilization, and potassium solubilization. Co-culture assays with *A. thaliana* and pot experiments on *P. praeruptorum* demonstrated that endophytic strains *A. welwitschiae* (G29) and *A. foetidus* (G30) exerted stable and efficient growth-promoting effects. These strains enhanced the growth of both *A. thaliana* and *P. praeruptorum* by regulating root development, photosynthesis, nutrient accumulation, and antioxidant enzyme systems.

These findings provide a theoretical basis for the green cultivation of *P. praeruptorum*, the development of microbial fertilizers, and the clarification of symbiotic interaction mechanisms between medicinal plants and their endophytes. Future work will focus on the correlation between microbial communities and secondary metabolites (e.g., coumarins) to strengthen the medicinal value orientation of this research.

## Data Availability

SRA data are available in NCBI (www.ncbi.nlm.nih.gov) under the accession number PRJNA1441879.

## Supplementary material

Supplementary Table S1 Identification results of fungal ITS gene sequences.

Supplementary Table S2 Identification results of bacterial 16S rRNA gene sequences.

Supplementary Table S3 Ratio of clear zone diameter to colony diameter (SI values) for screening dominant functional strains.

Supplementary Figure S1 Phylogenetic tree of endophytic fungi isolated from roots of *P. praeruptorum.*

Supplementary Figure S2 Phylogenetic tree of endophytic fungi isolated from stems of *P. praeruptorum.*

Supplementary Figure S3 Phylogenetic tree of endophytic fungi isolated from leaves of *P. praeruptorum.*

Supplementary Figure S4 Phylogenetic tree of endophytic bacteria associated with *P. praeruptorum.*

Supplementary Figure S5 Plant growth-promoting (PGP) traits of representative isolated strains.
