# Supplementary figures and images for: Spatiotemporal dynamics of culturable endophytic microorganisms in Peucedanum praeruptorum Dunn and screening for plant growth-promoting strains
Source: Front Plant Sci. 2026 Apr 27;17:1811872. doi: 10.3389/fpls.2026.1811872 (PMC13158224; doi:10.3389/fpls.2026.1811872)

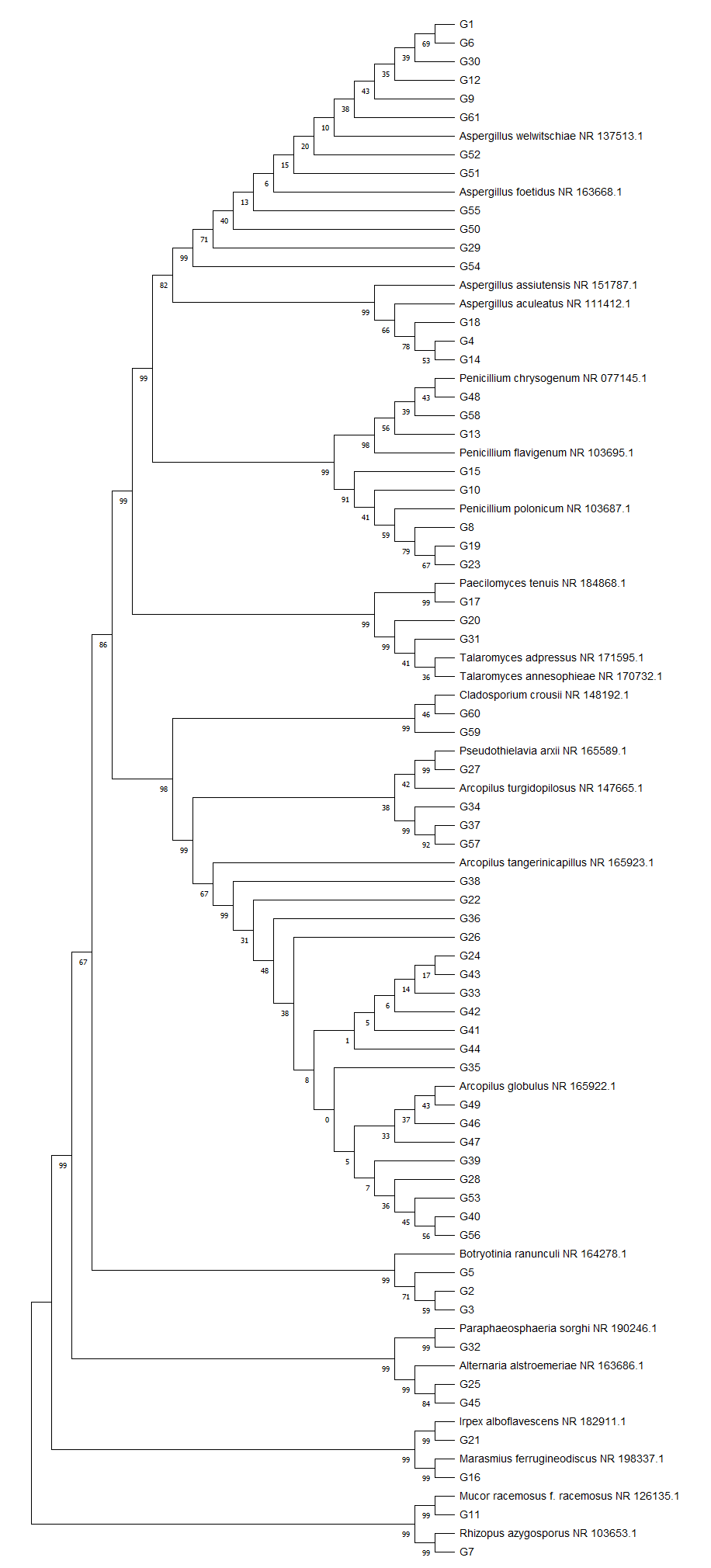

Supplement: Supplementary Figure S1 — Phylogenetic tree of endophytic fungi isolated from roots of P. praeruptorum. [file Image1.png]

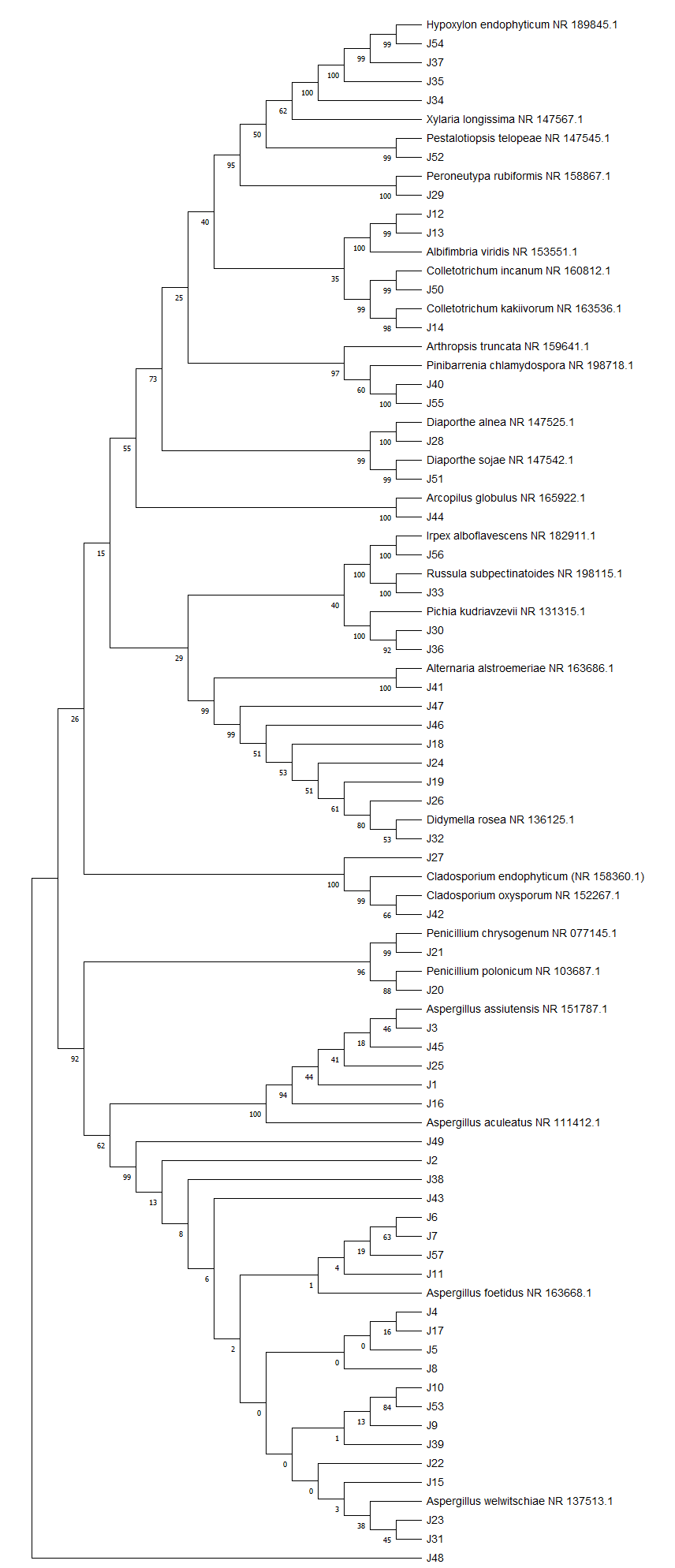

Supplement: Supplementary Figure S2 — Phylogenetic tree of endophytic fungi isolated from stems of P. praeruptorum. [file Image2.png]

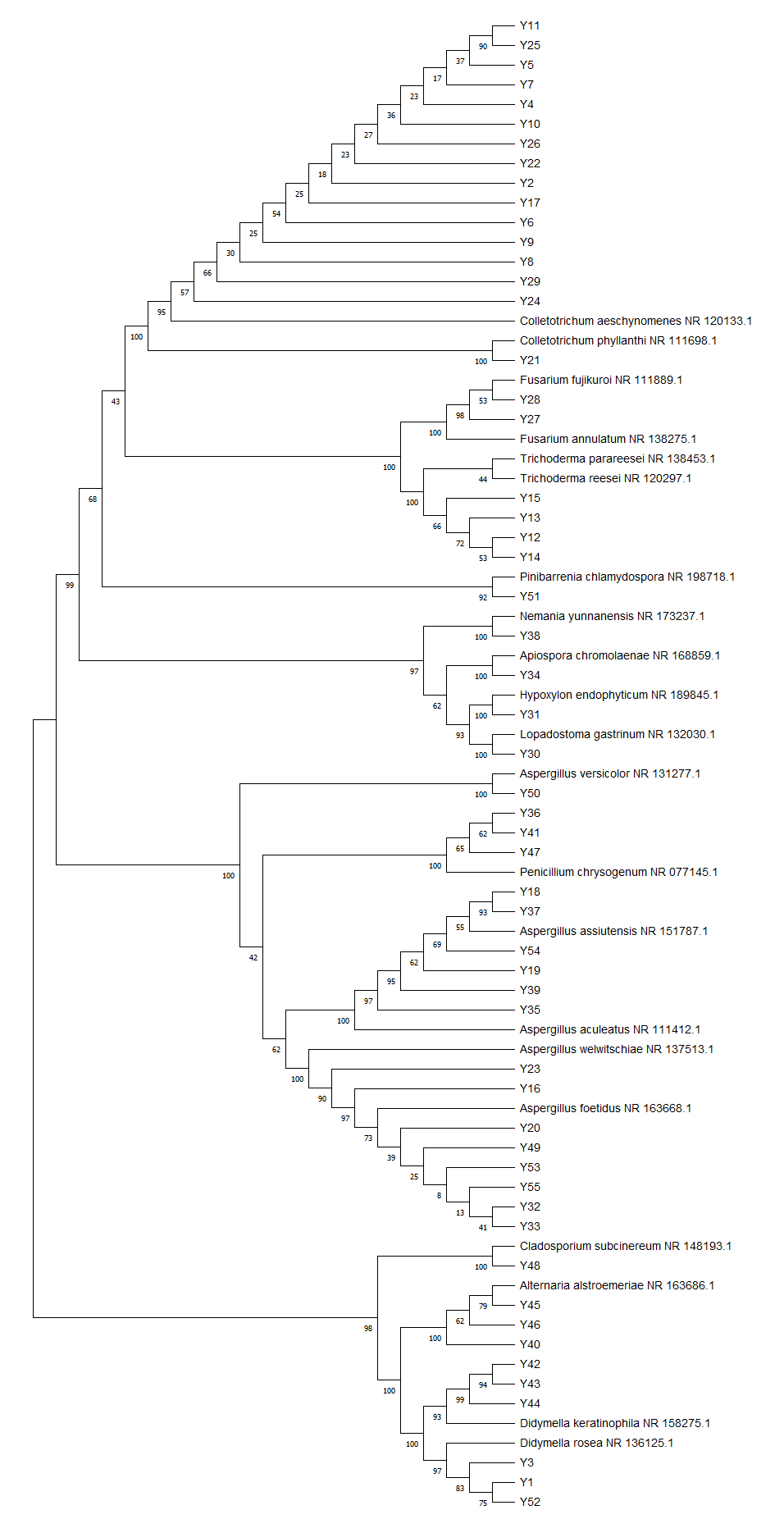

Supplement: Supplementary Figure S3 — Phylogenetic tree of endophytic fungi isolated from leaves of P. praeruptorum. [file Image3.png]

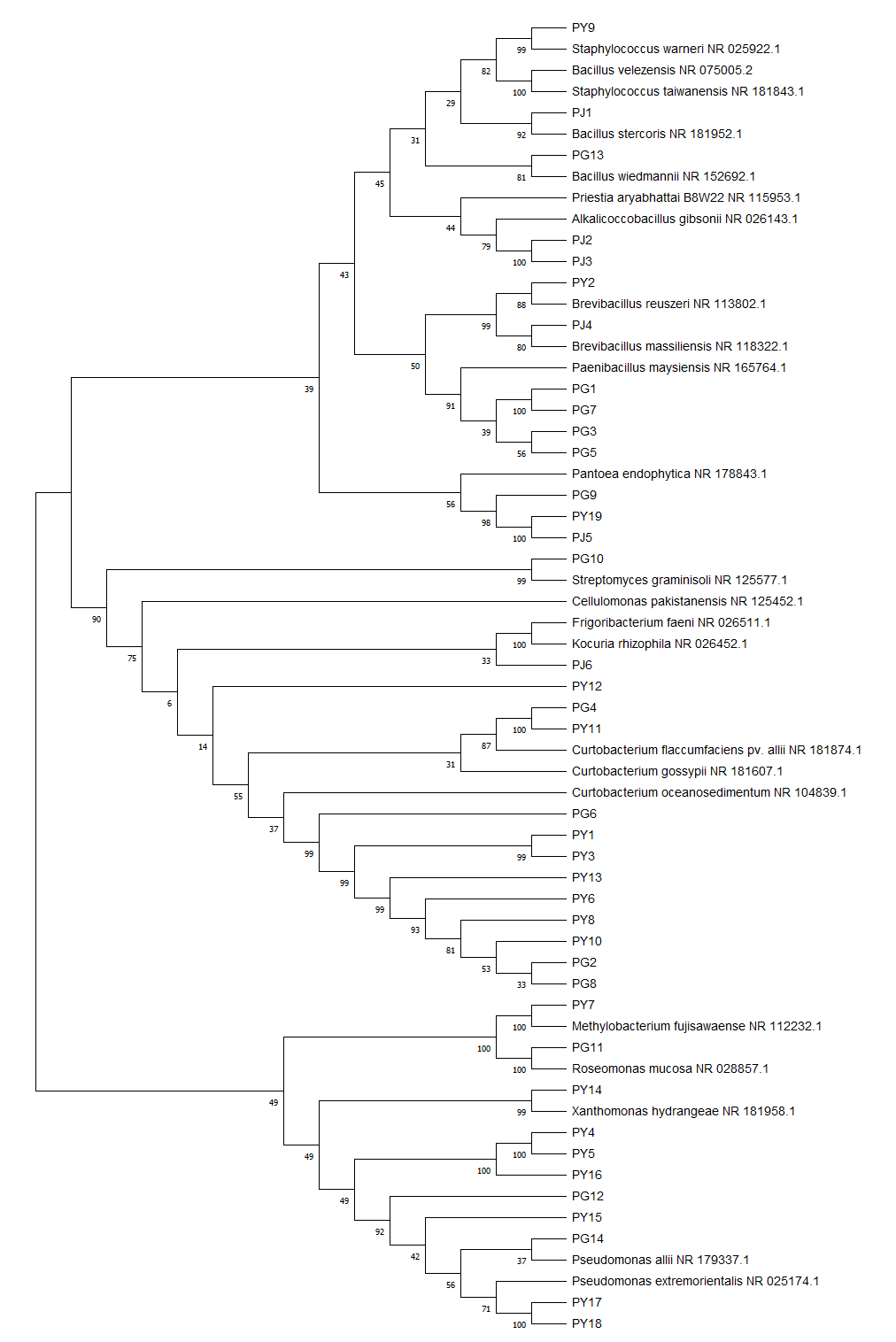

Supplement: Supplementary Figure S4 — Phylogenetic tree of endophytic bacteria associated with P. praeruptorum. [file Image4.png]

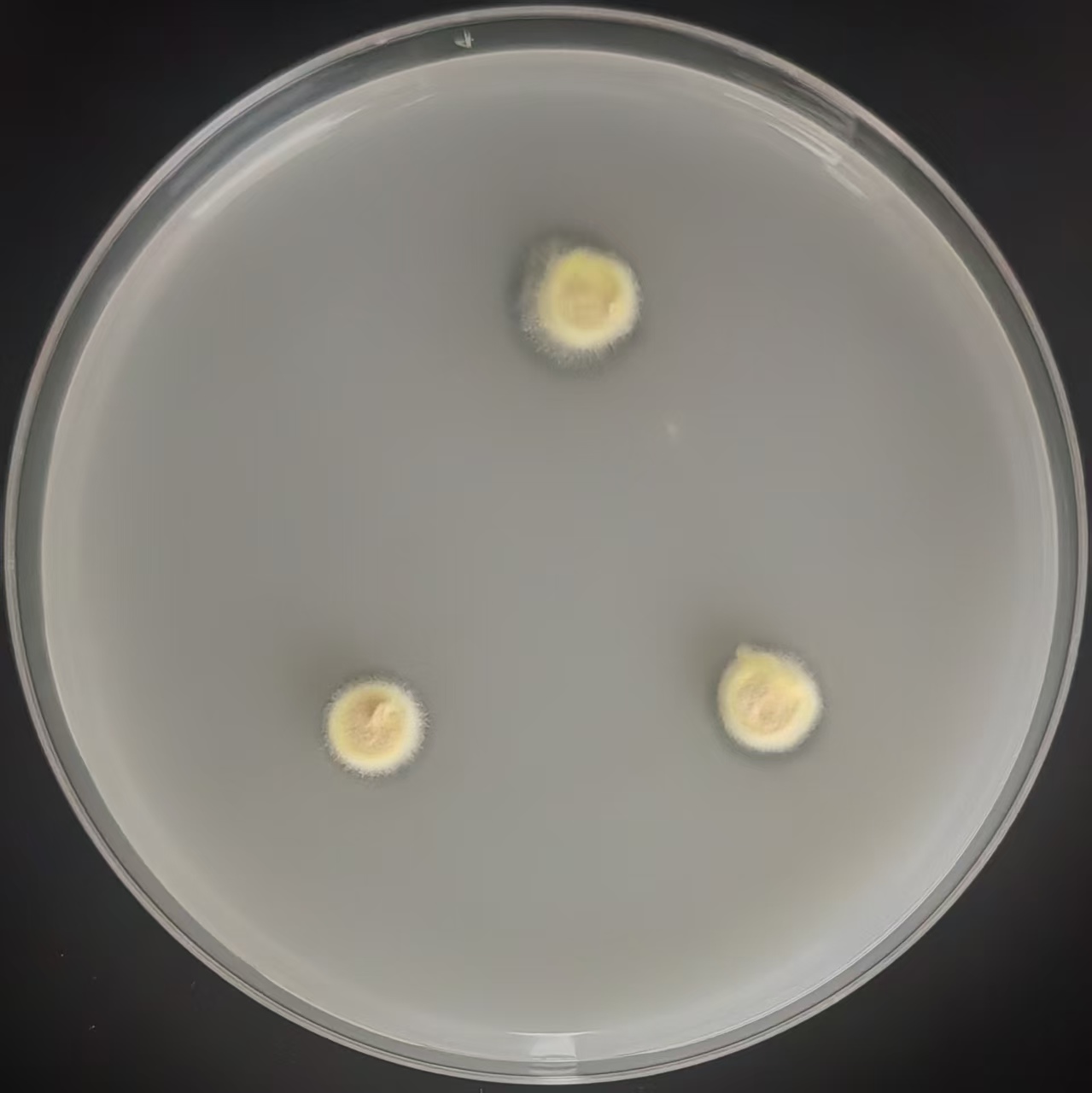

Supplement: Supplementary Figure S5 — Plant growth-promoting (PGP) traits of representative isolated strains. [file DataSheet1.zip › Figure S5/inorganic phosphate solubilization-G31.jpg]

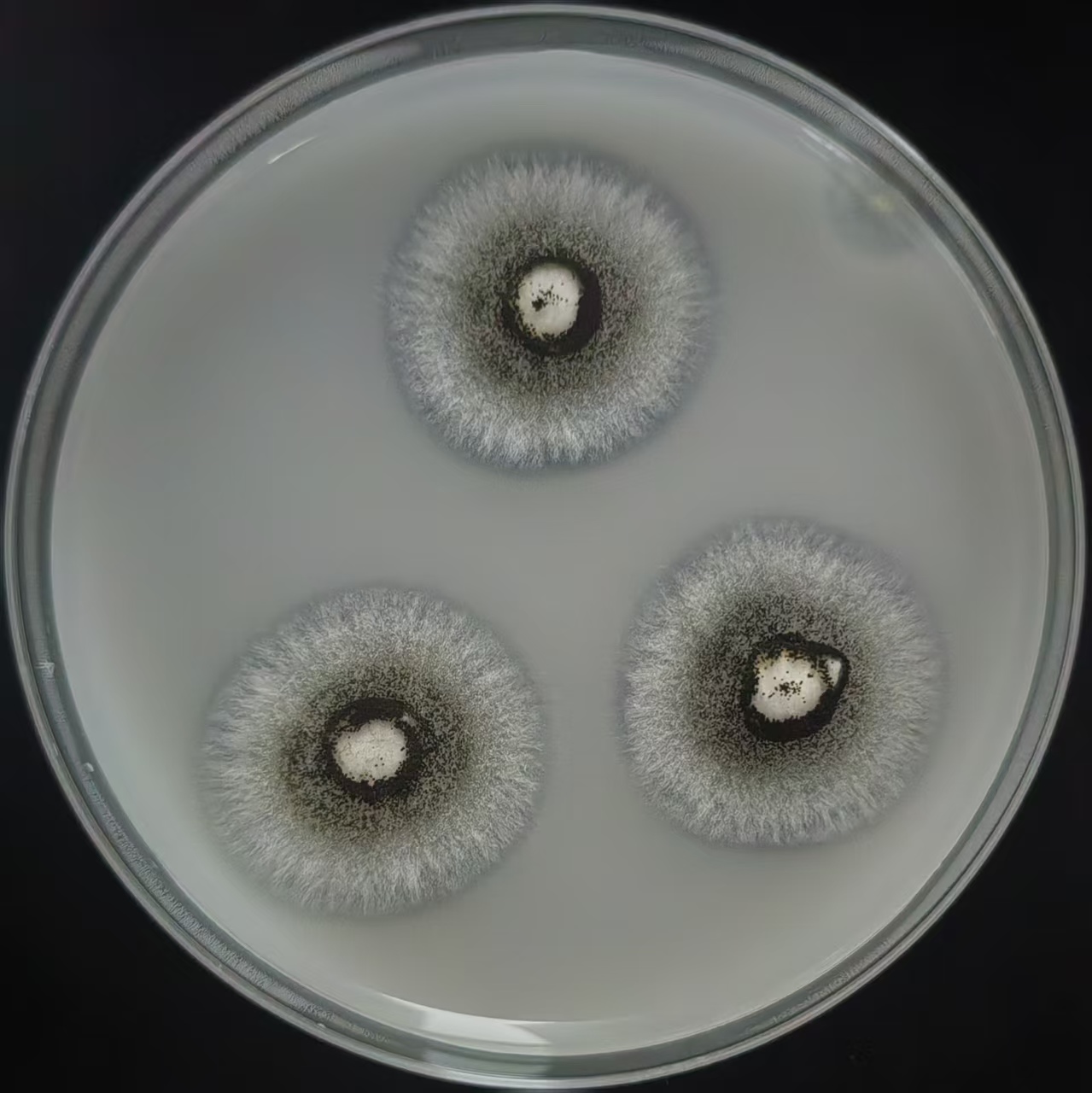

Supplement: Supplementary Figure S5 — Plant growth-promoting (PGP) traits of representative isolated strains. [file DataSheet1.zip › Figure S5/inorganic phosphate solubilization-J8.jpg]

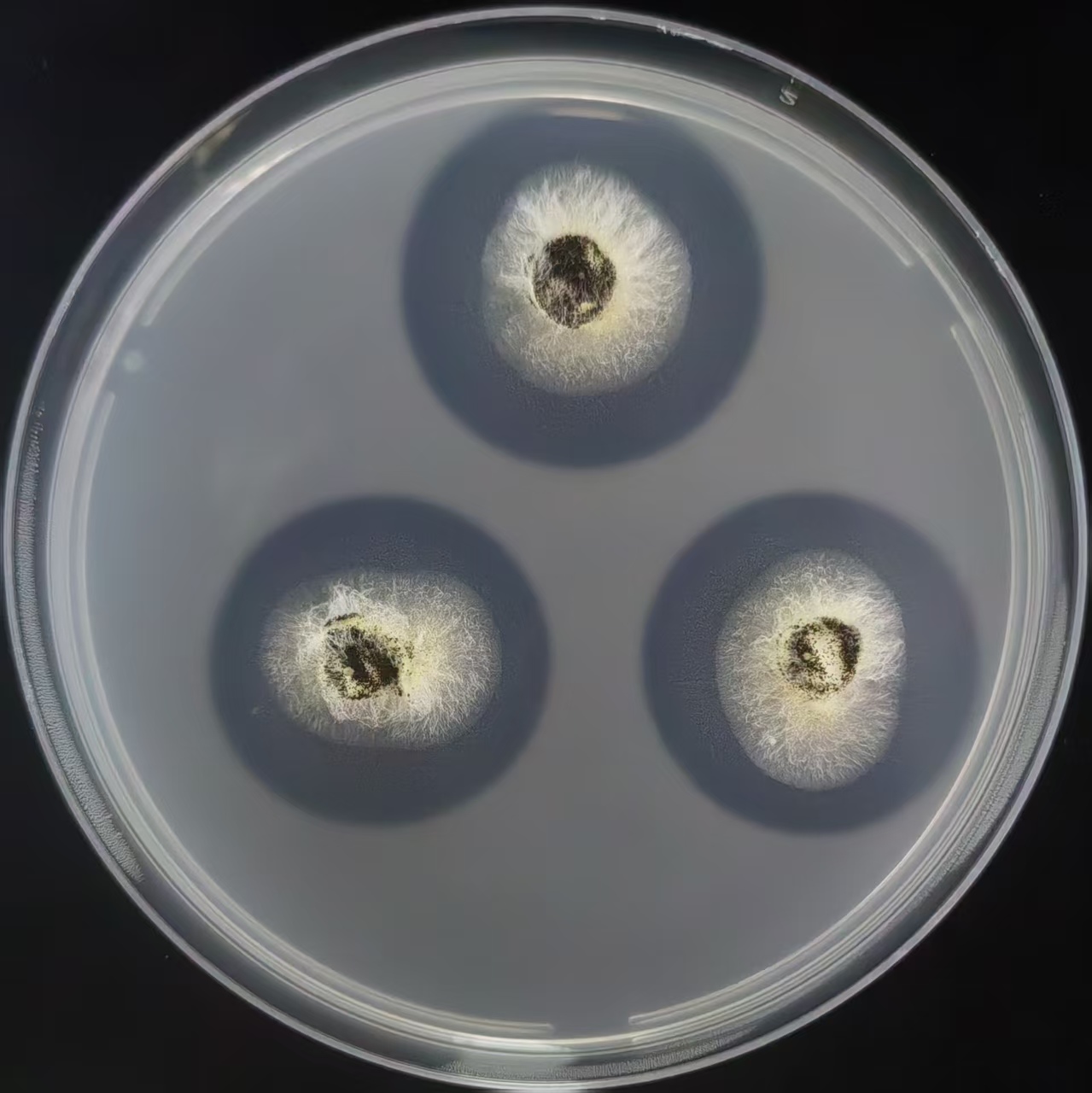

Supplement: Supplementary Figure S5 — Plant growth-promoting (PGP) traits of representative isolated strains. [file DataSheet1.zip › Figure S5/organic phosphate solubilization-G29.jpg]

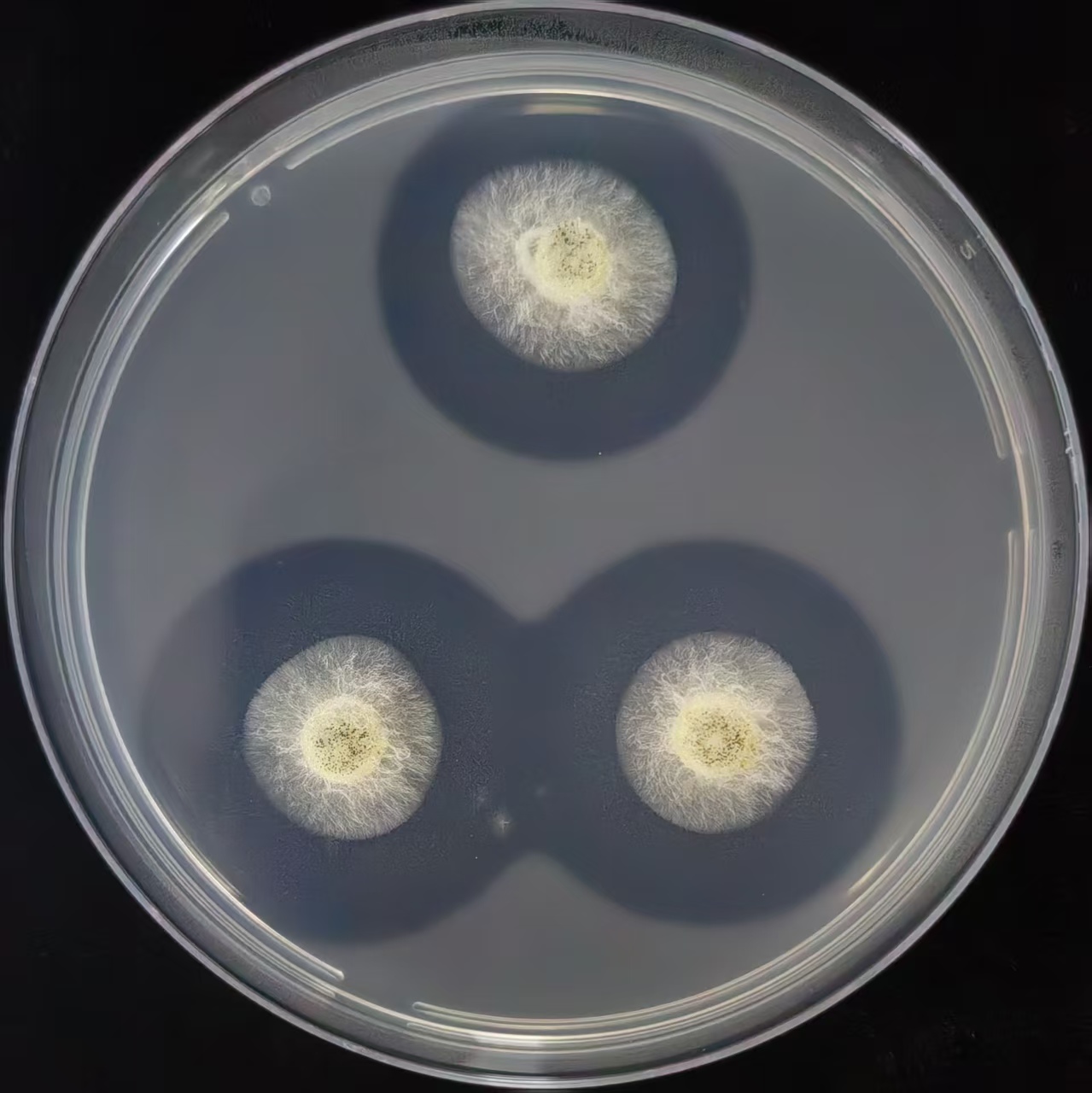

Supplement: Supplementary Figure S5 — Plant growth-promoting (PGP) traits of representative isolated strains. [file DataSheet1.zip › Figure S5/organic phosphate solubilization-G30.jpg]

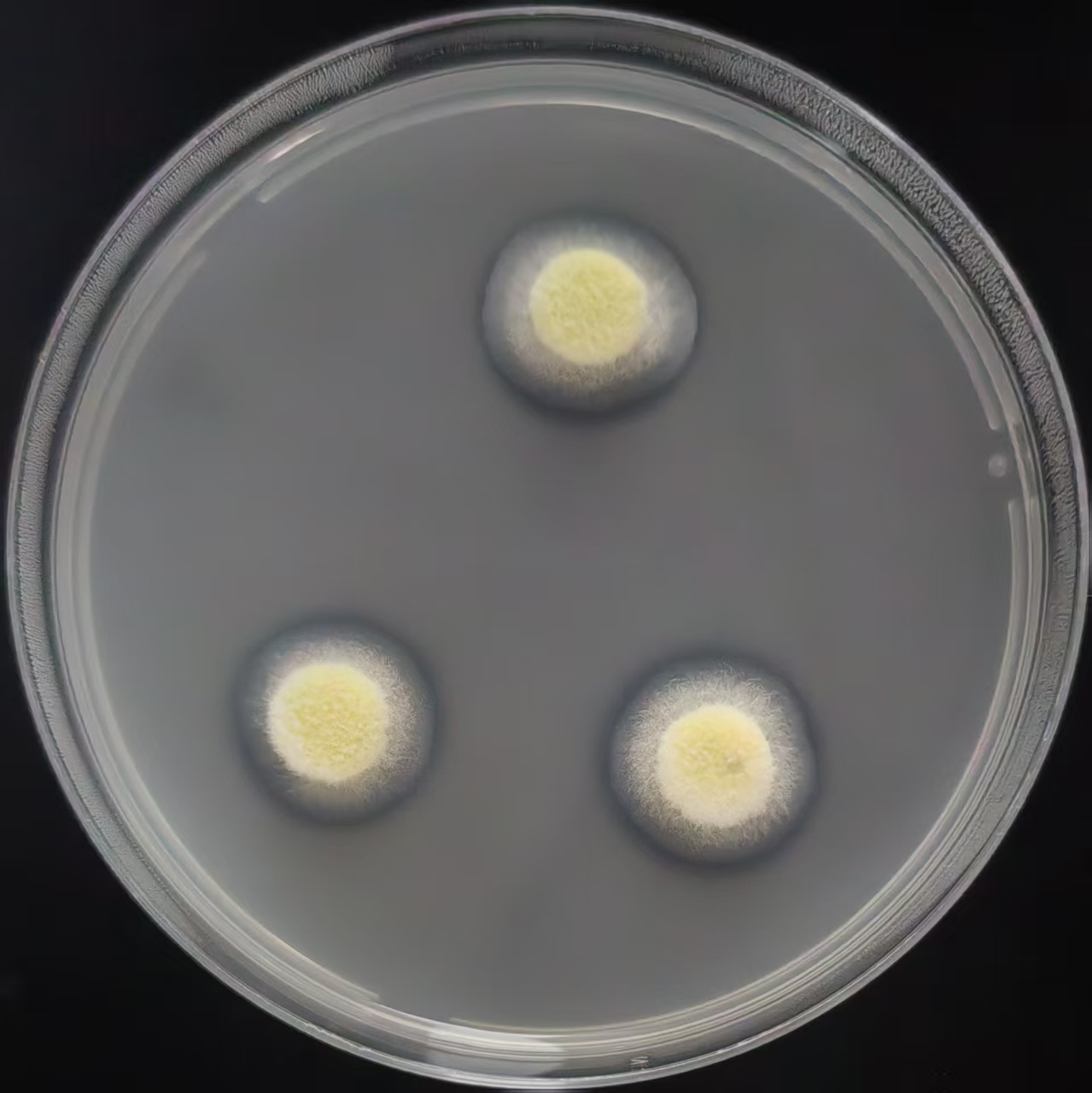

Supplement: Supplementary Figure S5 — Plant growth-promoting (PGP) traits of representative isolated strains. [file DataSheet1.zip › Figure S5/organic phosphate solubilization-G31.jpg]

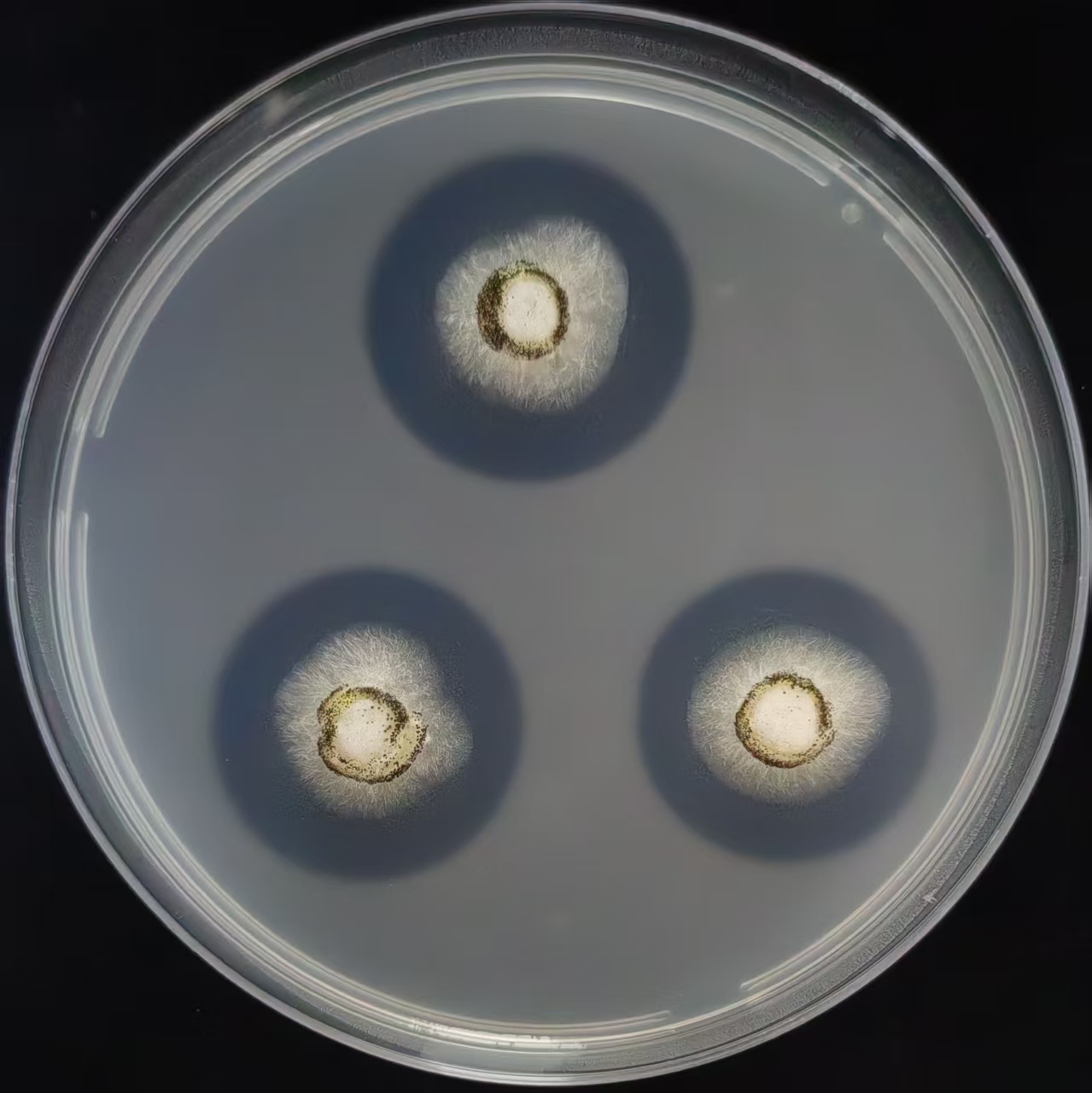

Supplement: Supplementary Figure S5 — Plant growth-promoting (PGP) traits of representative isolated strains. [file DataSheet1.zip › Figure S5/organic phosphate solubilization-J8.jpg]

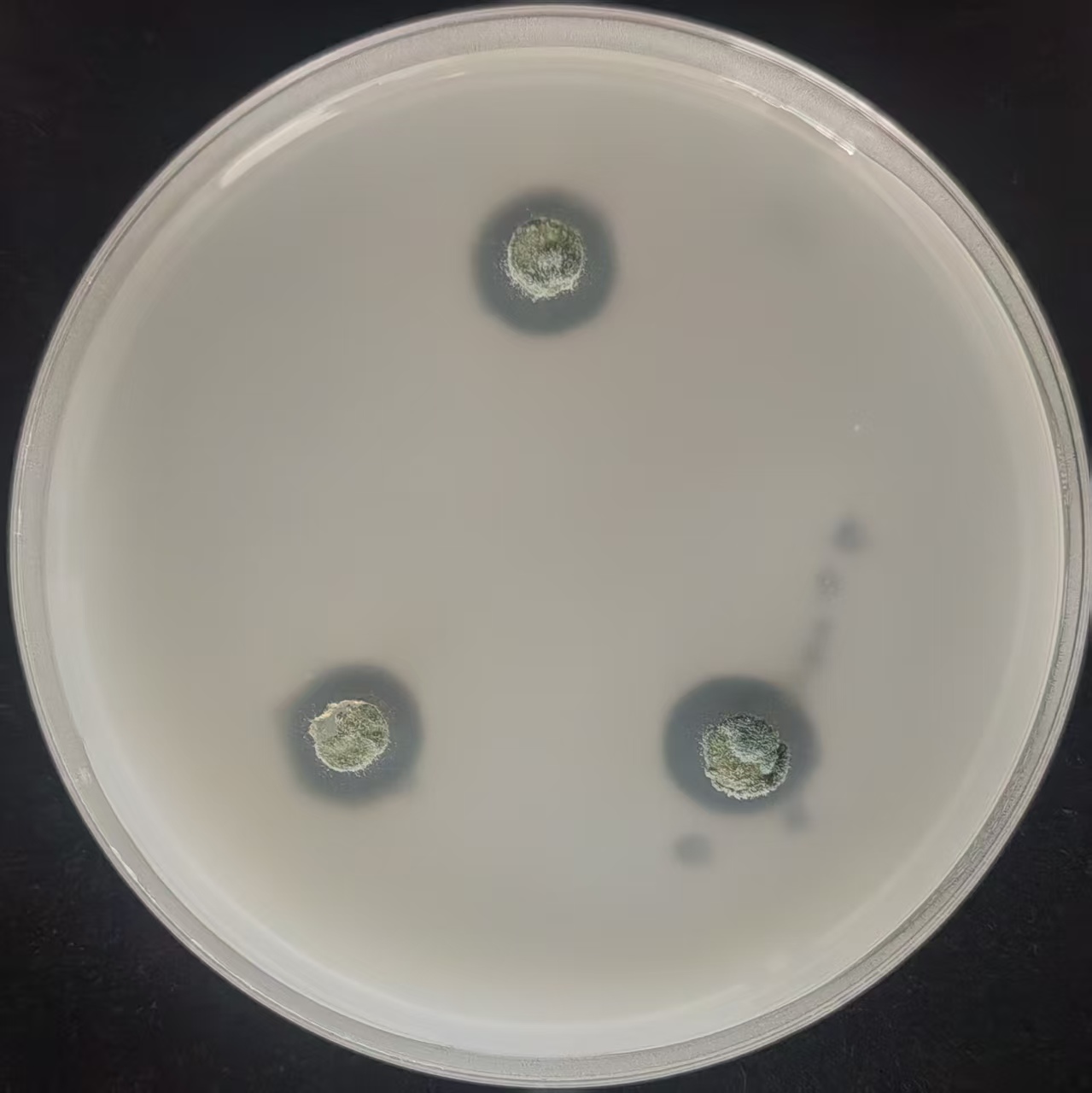

Supplement: Supplementary Figure S5 — Plant growth-promoting (PGP) traits of representative isolated strains. [file DataSheet1.zip › Figure S5/potassium solubilization-G16.jpg]

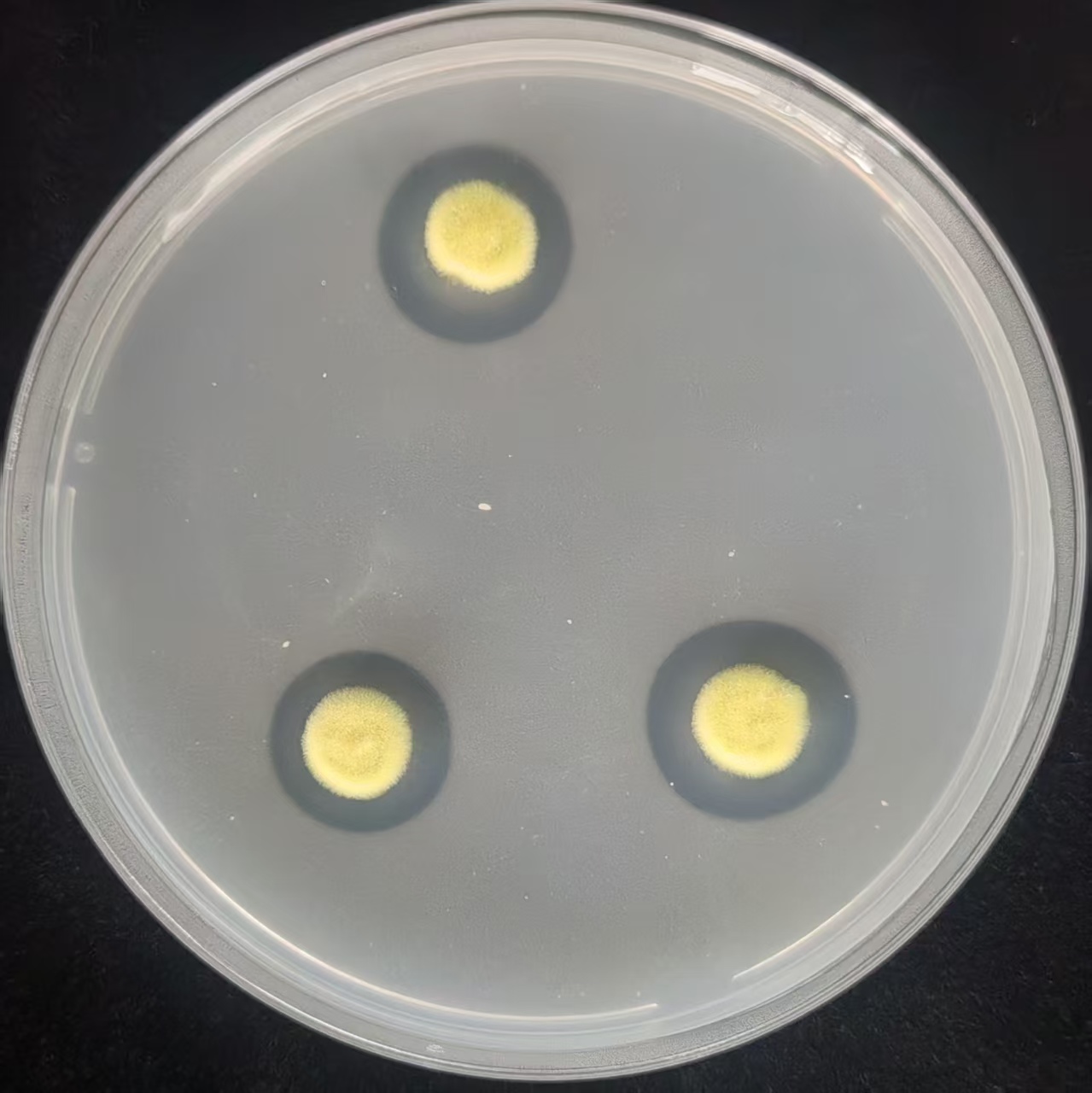

Supplement: Supplementary Figure S5 — Plant growth-promoting (PGP) traits of representative isolated strains. [file DataSheet1.zip › Figure S5/potassium solubilization-G31.jpg]

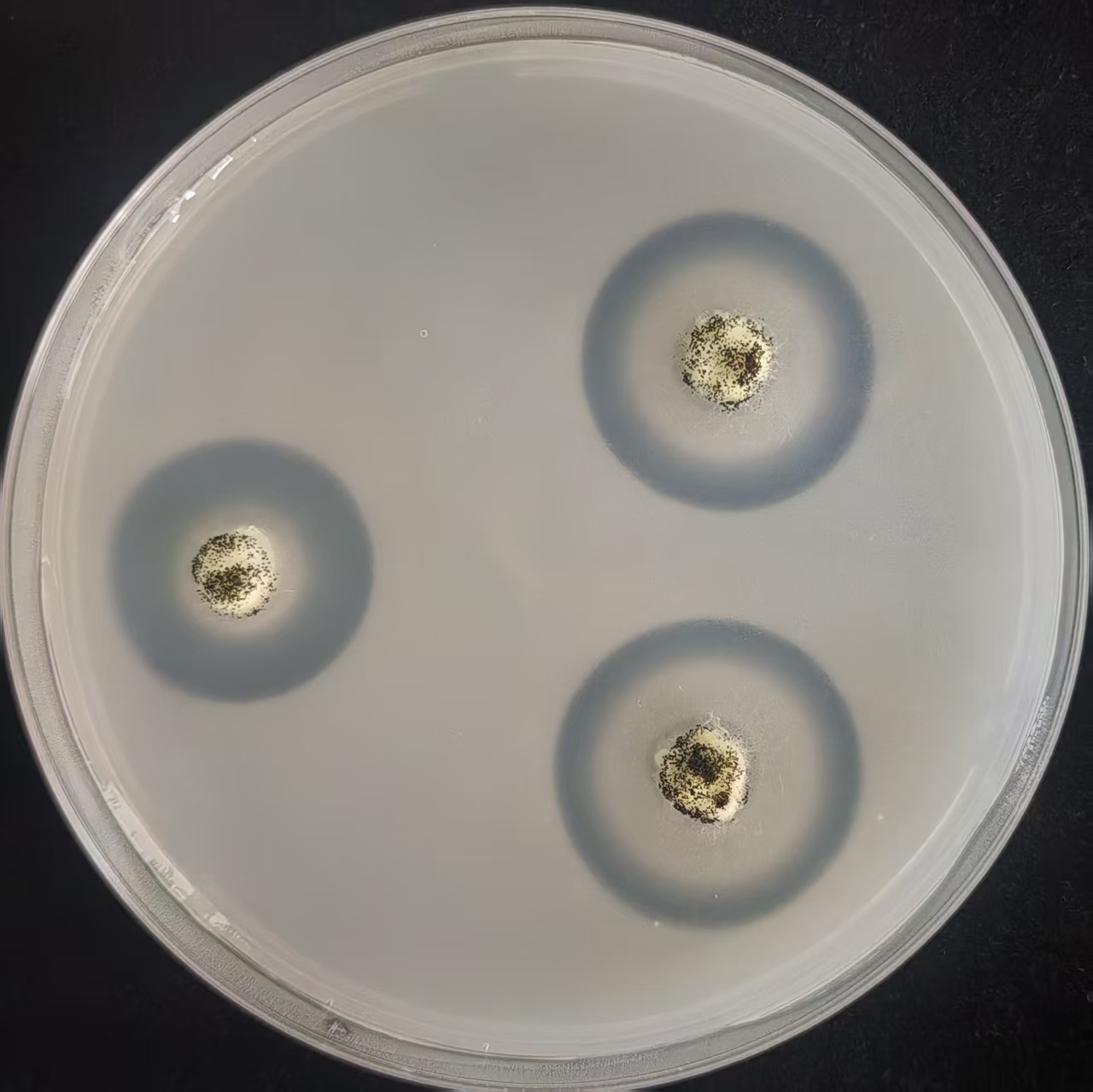

Supplement: Supplementary Figure S5 — Plant growth-promoting (PGP) traits of representative isolated strains. [file DataSheet1.zip › Figure S5/potassium solubilization-G60.jpg]

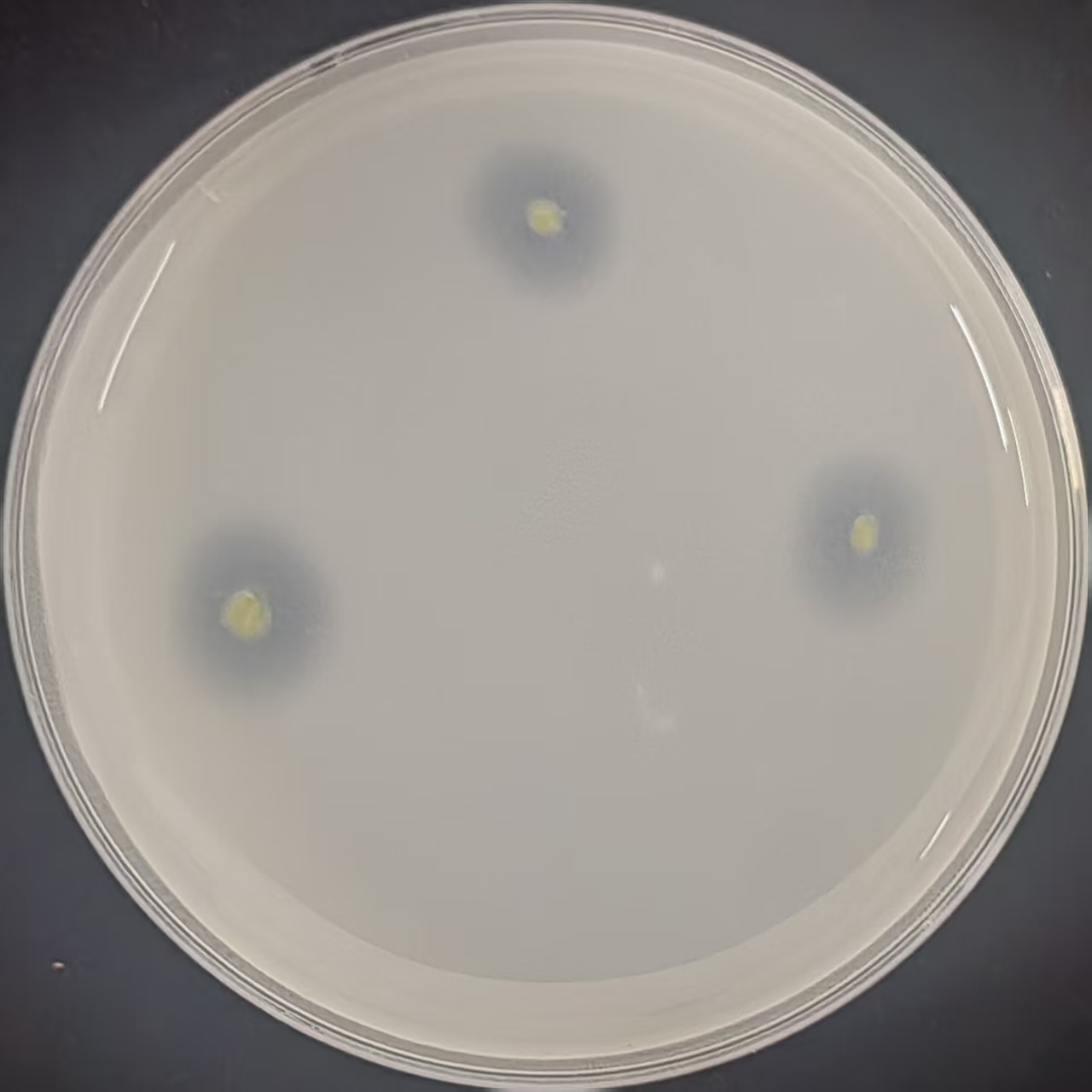

Supplement: Supplementary Figure S5 — Plant growth-promoting (PGP) traits of representative isolated strains. [file DataSheet1.zip › Figure S5/potassium solubilization-PY3.jpg]
